# Transcriptome Dynamics of Rooting Zone and Leaves during In Vitro Adventitious Root Formation in *Eucalyptus nitens*

**DOI:** 10.3390/plants11233301

**Published:** 2022-11-29

**Authors:** Paula G. Ayala, Raúl M. Acevedo, Claudia V. Luna, Máximo Rivarola, Cintia Acuña, Susana Marcucci Poltri, Ana M. González, Pedro A. Sansberro

**Affiliations:** 1 Laboratorio de Biotecnología Aplicada y Genómica Funcional, Instituto de Botánica del Nordeste (IBONE-CONICET), Facultad de Ciencias Agrarias, Universidad Nacional del Nordeste, Sgto. Cabral 2131, Corrientes W3402BKG, Argentina; 2 Mejoramiento Genético Forestal, INTA-EEA Concordia, CC 34, Concordia E3200AQK, Argentina; 3 Instituto de Biotecnología, CICVyA (INTA), Nicolas Repetto y de los Reseros s/n, Hurlingham, Buenos Aires B1686IGC, Argentina

**Keywords:** *Eucalyptus nitens*, adventitious rooting, transcriptome, auxins metabolism, recalcitrance

## Abstract

Wood properties and agronomic traits associated with fast growth and frost tolerance make *Eucalyptus nitens* a valuable forest alternative. However, the rapid age-related decline in the adventitious root (AR) formation (herein, meaning induction, initiation, and expression stages) limits its propagation. We analyzed transcriptomic profile variation in leaves and stem bases during AR induction of microcuttings to elucidate the molecular mechanisms involved in AR formation. In addition, we quantified expressions of candidate genes associated with recalcitrance. We delimited the ontogenic phases of root formation using histological techniques and *Scarecrow* and *Short-Root* expression quantification for RNA sequencing sample collection. We quantified the gene expressions associated with root meristem formation, auxin biosynthesis, perception, signaling, conjugation, and cytokinin signaling in shoots harvested from 2- to 36-month-old plants. After IBA treatment, 702 transcripts changed their expressions. Several were involved in hormone homeostasis and the signaling pathways that determine cell dedifferentiation, leading to root meristem formation. In part, the age-related decline in the rooting capacity is attributable to the increase in the *ARR1* gene expression, which negatively affects auxin homeostasis. The analysis of the transcriptomic variation in the leaves and rooting zones provided profuse information: (1) To elucidate the auxin metabolism; (2) to understand the hormonal and signaling processes involved; (3) to collect data associated with their recalcitrance.

## 1. Introduction

In forestry, vegetative propagation is extensively used to multiply plants with desirable traits obtained in breeding programs or selected from natural populations. Among the numerous techniques available, rooting stem cuttings from selected genotypes is the most efficient and cost-effective method to produce large quantities of identical plants [1]. However, not all tree species are responsive to this type of propagation. Furthermore, massive losses can occur if the cuttings do not form adventitious roots (ARs) or if they produce poor-quality root systems. 

Adventitious root formation is a post-embryonic organogenic process in which roots are induced from determined cells that have not been specified to develop roots. The de novo induction of organs or whole organisms in an ectopic location, such as the appearance of roots from stem cuttings, comprises the induction of meristems from adult differentiated somatic cells as a result of two stimulating processes: Wounding responses at the cutting base and signal network isolation from the whole plant [2,3]. Consequently, the ontogenic process embraces three successive but interdependent physiological phases: Induction (the period preceding any histological event), initiation (cell divisions leading to the formation of internal root meristems), and expression (the development of the typical dome-shape structures, the internal growth of root primordia, and root emergence) [4]. In this context, the role of auxins as master regulators has been well established [4,5], and researchers have recently reviewed the genetic [6], molecular [7,8,9], and physiological [1] aspects of adventitious root formation. Moreover, the age of the cutting donor plant also affects the rooting ability, and researchers have made considerable efforts to elucidate the biochemical and molecular mechanisms involved [10]. 

*Eucalyptus nitens* (Deane and Maiden) Maiden, which is commonly known as shining gum, is primarily planted for kraft pulpwood, and to a lesser extent, for lumber and veneer production, in the high-rainfall temperate regions of Australia, Chile, New Zealand, and Southern Africa [11,12]. *E. nitens* is typically propagated by seeds [13]; however, it requires long periods for seed maturation, which results in a low and irregular germination rate [14,15]. 

Vegetative propagation of this species using conventional [16] and tissue culture procedures [17,18,19,20] has been achieved, even though the wide-scale commercial application is limited. In effect, propagation is only possible if zygotic embryos or young seedlings are used as the explant sources instead of adult vegetative or reproductive-stage individuals. Furthermore, due to the strong recalcitrance character, *E. nitens* quickly decreases the adventitious rooting competence during ageing. This phenomenon is extended to other species, including *E. gunii* [21] and *E. globulus* [22,23].

To further understand the molecular mechanism of adventitious root formation, we used a transcriptomic approach in the present study to identify the genes associated with root formation of in vitro *E. nitens* shoots during root induction. Subsequently, we selected and quantified the expressions of candidate genes involved in the ageing loss of root competence. Three experimental design elements enhanced the clarity and relevance of our findings: (1) we took samples from leaves and rooting zones; (2) we defined sampling time by histological analysis and quantitative *Scarecrow* (*SCR*) and *Short-Root* (*SHR*) gene expressions; (3) we used RNA sequencing to identify global gene expression profiles.

## 2. Results

**Figure 1:**
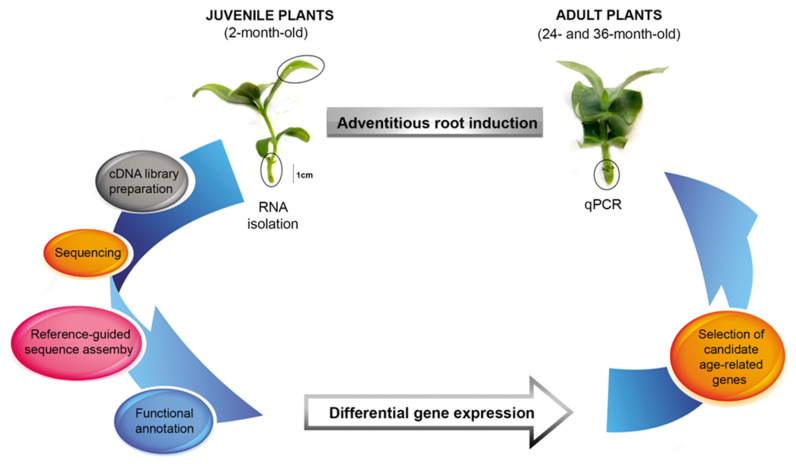
Schematic diagram of the experimental design to study the transcriptomic profile variation and age-related gene quantification during the induction phase of *E. nitens* root formation.

In Figure 1, we summarize the experimental strategy for determining the differential gene expressions and selecting the ageing candidate genes. We used a rooting procedure based on an exogenous application of indole-3-butyric acid (IBA) [20]. IBA, a natural precursor of IAA, has been widely used in woody plant propagation because it is more stable and effective than IAA in promoting adventitious root development, primarily due to its higher resistance to light-induced degradation. 

Initially, we time delimited the ontogenic phases of adventitious root formation through the histological analysis and quantification of the *SCR* and *SHR* expressions. We isolated the total RNA from the leaves and basal stems (rooting zone) of IBA-treated shoots harvested early in the induction phase from juvenile plants. Then, we synthesized the cDNA from the mRNA for use in the RNA-Seq and qPCR experiments. We obtained the transcriptomes by de novo assembly guided by the *E. grandis* reference genome. We annotated the transcripts and calculated their expression levels to find the differentially expressed genes between the two sample conditions. Finally, we quantified the expressions of the candidate age-related genes in shoots from adult plants during the adventitious root induction.

### 2.1. Ontogeny of In Vitro Root Formation

**Figure 2:**
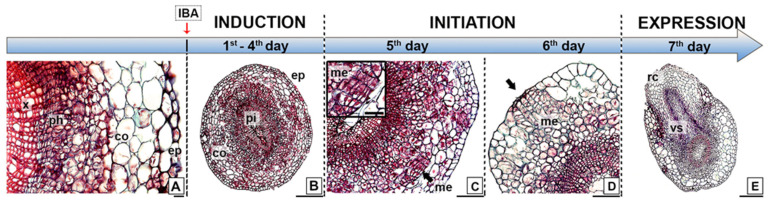
Ontogeny of in vitro root formation in juvenile shoots of *E. nitens*. (**A**) Transversal section of the basal stem, displaying a typical anatomical structure; (**B**) transversal section of the rooting zone after four days from the IBA treatment; (**C**) cell divisions nearest the periphery of cortical parenchyma; (**D**) small meristematic cells group leading root meristem formation; and (**E**) root emergence through the stem epidermis. Ref. co, cortex; ep, epidermis; me, meristemoids; ph, phloem; pi, pith; rc, root cap; x, xylem; vs, vascular system. Bars indicate 20 µm (**A**), 50 µm (**C**), 100 µm (**D**), and 200 µm (**E**).

Adventitious root formation by the microshoots of *E. nitens* progressed via a direct rooting pattern (Figure 2). We observed no structural changes in cell morphology during the first four days after IBA application (Figure 2A,B). In the transversal section, the basal zone of the stem displayed a typical anatomical structure with xylem and phloem, and the presence of a vascular cambium that initiates secondary growth. However, it did not develop a cork cambium or peridermis. Thus, it still had an epidermis and cortex. The epidermis was formed by a single cell layer, whereas several layers of large parenchymatous cells comprised the cortex. We observed a continuing starch grain accumulation during this induction period, which preceded active cell division. On the fifth day, the cells that were randomly distributed in the cortex and sub-epidermal tissues reacquired the meristematic characteristics. Consequently, we could readily observe cell divisions nearest to the periphery of the cortical parenchyma, which led to the initiation phase of the root formation and resulted in small meristematic groups with relatively large nuclei and dense cytoplasms (Figure 2C,D). On the sixth day, we observed that the typical dome-shaped apex, protected by the root cap, was apparent. Finally, the roots emerged through the epidermis on the seventh day, starting the expression phase (Figure 2E). At this time, vascular connections were established between the adventitious root and the microshoot.

### 2.2. Quantification of SCARECROW and SHORT-ROOT Gene Expression

**Figure 3:**
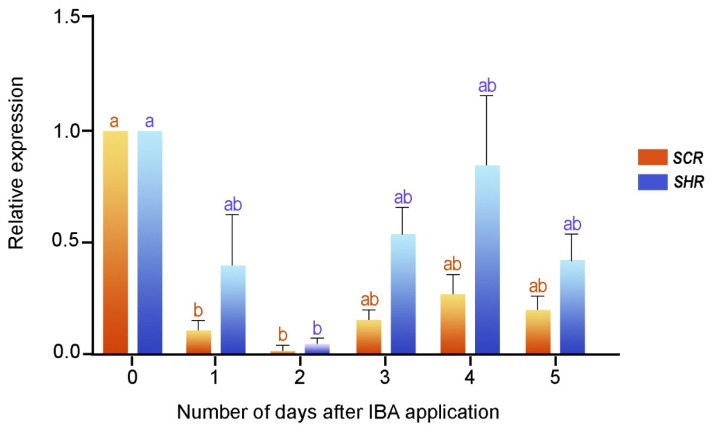
*SCR* and *SHR* gene expressions during the early step of adventitious root formation in two-month-old microshoots. Each column denotes the means of three replicates ± SEM Significant differences (*p* < 0.05) are marked using lowercase letters.

To validate the induction phase of the root formation, delimited by the histological analysis, we examined the expression profiles of the *SCR* and *SHR* genes in the rooting-competent microshoots of *E. nitens*. We performed reverse transcription followed by real-time quantitative PCR (RT-qPCR) for the first five days after the IBA treatment, which corresponded to the time necessary for root determination (see Section 2.1). We extracted the total RNA at different times from the IBA-treated cuttings, and we expressed the results as relative values to time zero (Figure 3). The expression profiles of both genes were comparable and significantly decreased (*p* < 0.01) within the first two days after the IBA application. *SHR* and *SCR* expressions were slightly induced on day three, increased on day four, and decreased on day six. According to these results, the *SHR* and *SCR* transcripts were induced in the rooting-competent microshoots before cell division and confirmed the induction stage of root formation. 

### 2.3. Transcriptome Sequencing and Assembly

We extracted high-quality RNA from the *E. nitens* leaves and stem bases before (S0) and three/four days (S3/4) after the IBA treatment (Supplementary Materials, Figure S1), taking each sample in triplicate (A, B, and C pools). Thus, we constructed and subsequently sequenced 12 cDNA libraries on the NextSeq500 MO Illumina^®^ platform. A total of 177,643,614 paired-end reads (2 × 151 bp) were generated, with a 49% GC content. We stored the raw sequence in the Sequence Read Archive of The National Center for Biotechnology Information (NCBI), and they are identified with the SRA accession number PRJNA891431.

**Figure 4:**
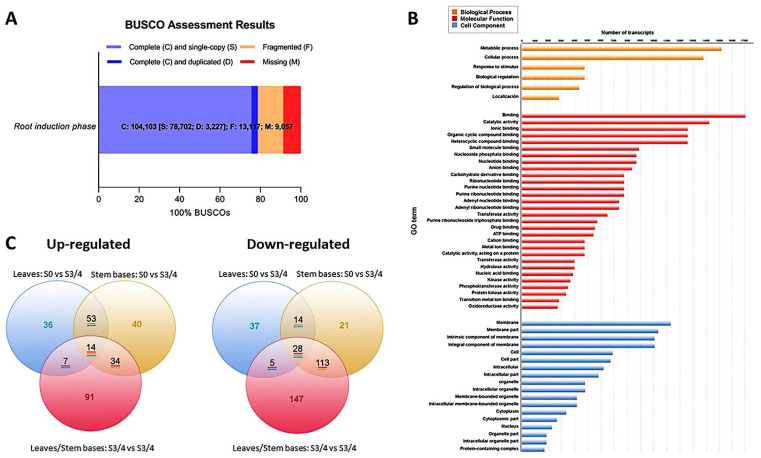
Data integrity assessment and annotation analysis. (**A**) BUSCO completeness assessment of the whole transcriptome data. The numbers indicate the absolute amount of complete and single-copy, complete and duplicated, fragmented, and missing BUSCO genes. (**B**) GO terms from leaves and stem bases transcripts. (**C**) Venn diagrams of differentially expressed genes in leaves and stem bases during root induction. The number of transcripts common in two or more comparative samples is enclosed in the overlapping portion of the circles.

After filtering, we only used the high-quality and clean reads in a genome-guided RNA-Seq assembly strategy. From these filtered reads, we mapped 64.92% to the *E. grandis* genome; however, 35.08% remained unaligned. Trinity software assembled 132,379 transcripts, and the completeness of this transcriptome was assessed by BUSCO (benchmarked universal single-copy orthologs) [24]. The software classified 104,103 from the total assembled transcripts as follows (Figure 4A): 78,702 (75.6%) were single-copy genes, and 3227 (3.1%) were duplicated genes. Another 9057 (8.7%) and 13,117 (12.6%) genes were missing and fragmented, respectively.

### 2.4. Functional Annotation and Classification

We filtered the transcripts with a deficient number of reads to avoid possible problems in the later interpretation. We only considered transcripts that presented counts per million reads mapped greater than 1 (CPM > 1) and that were present in at least six libraries. Thirty-four thousand seven hundred-one transcripts met these conditions, and we utilized them for further analysis.

The Gene Ontology (GO) database created the functional annotations of the assembled transcripts. Of the 34,701 selected transcripts, Blast2GO assigned GO terms to 30,683 transcripts, which it classified into three functional categories: Biological processes, molecular functions, and cellular components (Figure 4B). The genes involved in metabolic and cellular processes were more abundant than those involved in the biological processes. For example, in the “molecular functions” category, the most represented groups were “binding” and “catalytic activity”, while “membrane components”, “cellular part”, and “organelle” groups were more representative of the “cellular components”.

Finally, we used MapMan diagrams to generate an overview of the variation in the transcriptomic profiles modulated by the morphogenic process and linked to hormones, signaling pathways, and redox (Supplementary Materials, Figure S2). We determined the transcriptional changes by comparing the leaf and stem base data from two specific time points (S0 and S3/4). We created a Venn chart to display the number of differentially expressed transcripts (DETs) in each group (Figure 4C). We considered transcripts with a fold change (FC) ≥ 2 in their expressions between treatments and false discovery rates (FDR) < 0.05 to be differentially expressed. 

**Figure 5:**
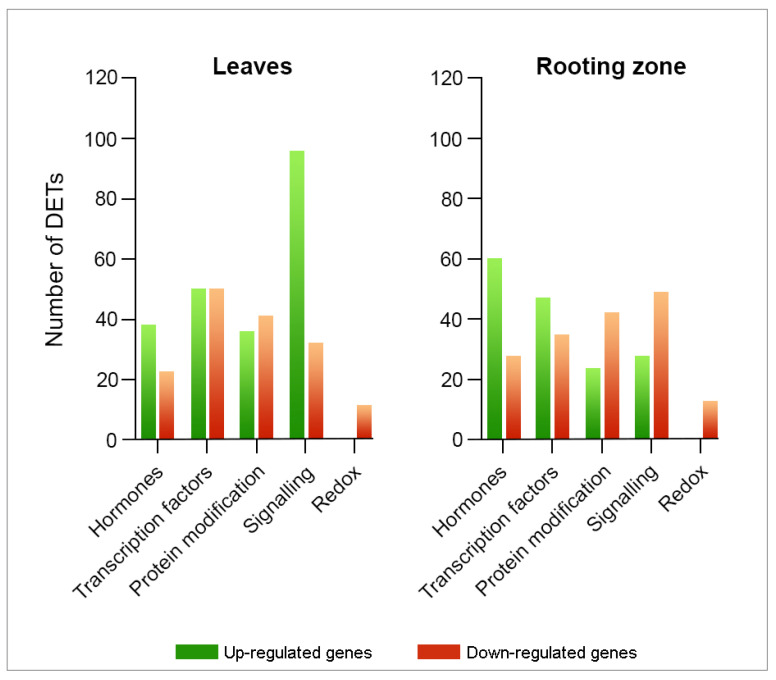
Transcriptomic profile modifications linked to hormone metabolism, signaling, and redox in *E. nitens* leaves and stem bases after three/four days of IBA-treatment. Fold-change ≥ 2 and FDR < 0.05.

We determined 376 DETs in the leaves (Figure 5) and linked hormone metabolism (60 DETs), transcription factors (100 DETs), and signaling processes (128 DETs). Likewise, we highlighted transcripts encoding kinases, calcium-mediated signaling and light receptors. At the same time, we recognized the differential expressions of transcripts associated with redox processes, including those encoding thioredoxins, glutaredoxins, and heme proteins. We identified 326 DETs in the rooting zone associated with hormone metabolism (88 DETs), transcription factors (82 DETs), and signaling processes (77 DETs), including kinases, calcium signaling, G proteins, and light receptors. Likewise, we identified DETs encoding thioredoxins, glutathione/ascorbate, glutaredoxins and heme proteins. 

### 2.5. Confirmation of Gene Expression by RT-qPCR Analysis

**Figure 6:**
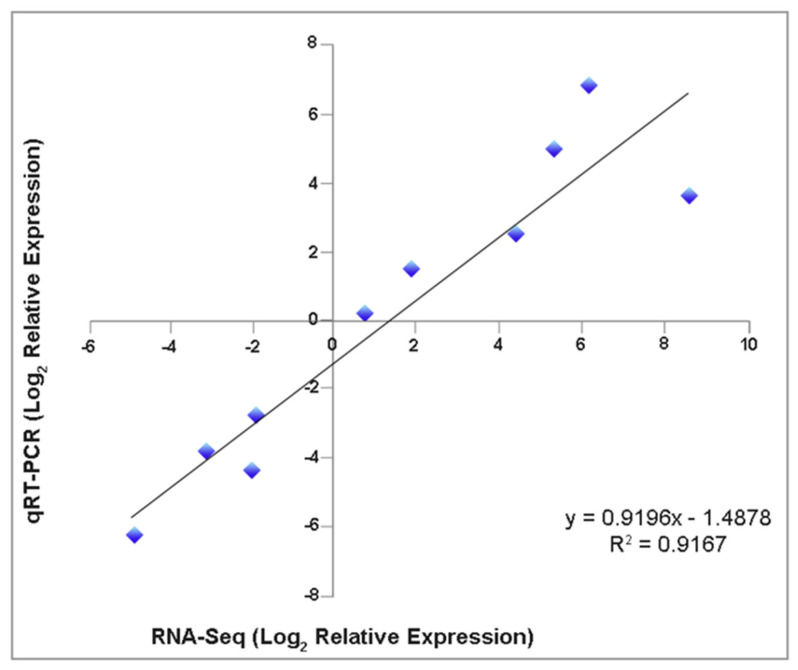
Expression level correlation from RNA–seq analysis and reverse transcription followed by quantitative real-time PCR (RT–qPCR). Ten randomly selected DETs were compared by both techniques (three biological replicates). The *EF2* (*Translation Elongation Factor 2*) gene was used as an internal control.

To confirm the DETs that we detected through the RNA-Seq analysis, we conducted reverse transcription followed by real-time quantitative PCR on ten randomly selected DETs (Supplementary Materials, Table S1). As a result, the transcripts of *Endo1* (*endoglucanase 1*), *CytokDes3* (*cytokinin dehydrogenase*), *ERF003* (*ethylene-responsive transcription factor*), *GH3-1* (*Gretchen Hagen 3*), *ARF6* (*auxin response factors*), and *SCR* (*Scarecrow*) had increased expressions. At the same time, *ACO* (*1-aminocyclopropane-1-carboxylate oxidase*), *FBA* (*fructose-bisphosphate aldolase*), *ISC-like* (*isoprene synthase, chloroplastic*-like), and *CSACat8* (*cellulose synthase A catalytic 8*) had decreased expressions on day three/four after the IBA treatment. We further validated the changes in the gene expressions identified through the RNA-Seq by comparing the fold changes in the sequence reads with the fold changes determined by the RT-qPCR. The fold changes determined by both methods fell on a single-fitted straight line, with R^2^ = 0.92 (Figure 6). This concordance between the RNA-Seq and RT-qPCR data confirmed the authenticity of the DETs and validated the findings from our transcriptome study.

### 2.6. Expressions of Age-Related Genes

**Figure 7:**
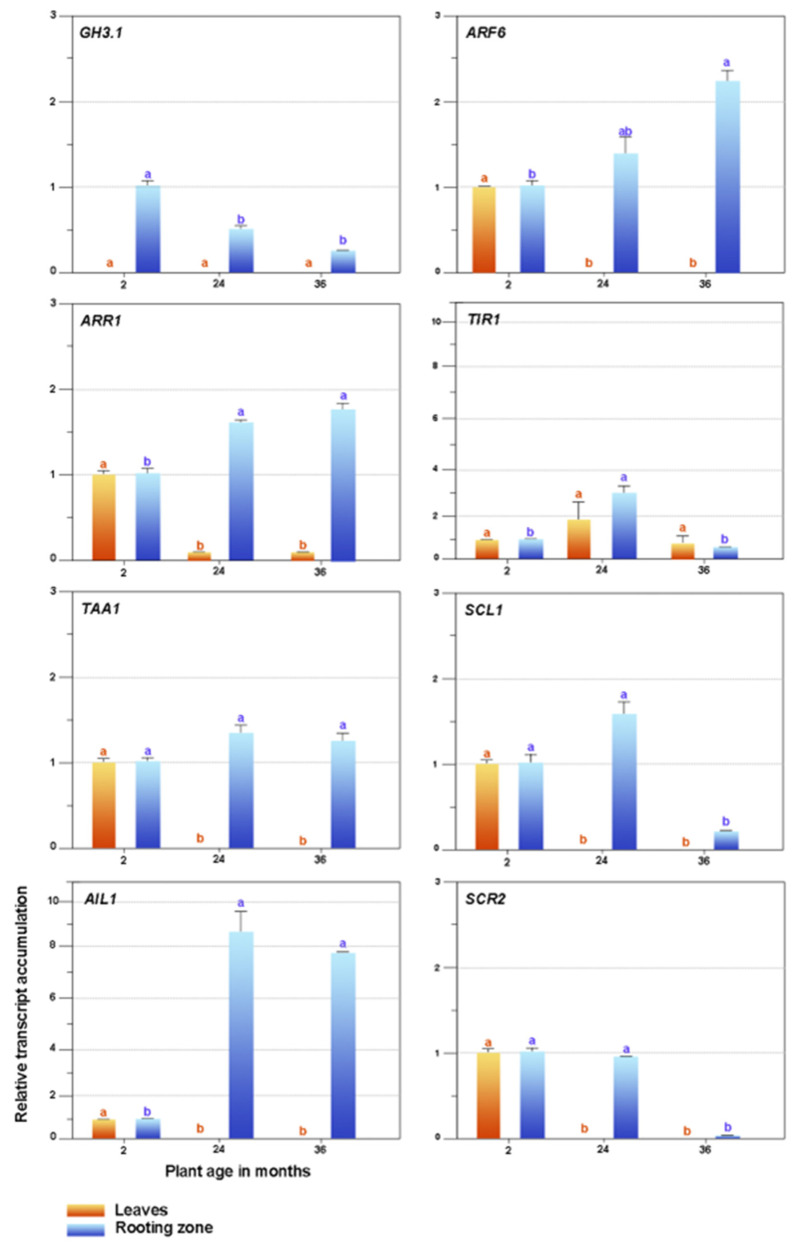
Expression patterns of candidate genes related to plant ageing and rooting competence. *GH3-1*, *Gretchen Hagen3*; *ARF6*, *auxin response factor*; *ARR1*, *Arabidopsis response regulator 1*; *TIR1*, *transport inhibitor response 1*; *TAA1, tryptophan aminotransferase in Arabidopsis 1*; *SCL1, Scarecrow*-like *1*; *AIL1*, *aintegmenta*-like 1; *SCR2*, *Scarecrow-2*. Bars indicate mean ± SEM Significant differences (*p* < 0.05) are marked using lowercase letters. The EF2 gene was used as an internal control.

We present the expressions of selected genes in the leaves and stem bases of the in vitro established shoots collected from plants of different ages in Figure 7. The *ARR1* expression significantly diminished in the leaves of the adult shoots. *TIR1* remained constant, while we observed that the expressions of *ARF6*, *TAA1*, *SCL1*, *AIL1*, and *SCR2* were only visible in two-month explants. We did not detect *GH3.1* transcripts. 

The expressions of the *SCR2* and *SCL1* genes substantially decreased in the rooting zones of the 36-month shoots, while conversely, the *ARR1* and *AIL1* expressions declined in the juvenile explants and increased in the 24- and 36-month-old shoots. Ageing did not affect the *TAA1* expression. *TIR1* presented a variable performance, *ARF6* increased due to age effects, and *GH3.1* was the maximum in juvenile shoots and declined in adult ones.

## 3. Discussion

### 3.1. Histological Determination of Root Induction Stage in Juvenile Shoots

The initial phase of adventitious root formation, called induction, is characterized as an anatomical lag phase devoid of cellular changes, during which the initial cell reprogramming occurs and comprises a plethora of molecular and biochemical modifications that precede visible morphological changes [4,7]. According to our results, this event occurred in the first four days after the hormonal treatment, showing high accumulation of starch grains at the shoot bases. This biochemical process has been associated with a coordinated increase in the number of mitochondria and other organelles [25]. Consequently, the first cellular divisions occurred after five days in the cortical parenchyma and formed small meristematic groups. This evidence is consistent with that observed in numerous woody species, including *E. globulus* [26], chestnut [27], apple [28], and olive [29], among others, in which the neoformation of adventitious roots arises from the cortical tissues.

Furthermore, the cutting excision from the donor plant modifies the plant hormone homeostasis in the isolated shoot, making auxins an effective inducer of AR formation [7]. Concurrently, transcriptional regulatory networks may function as developmental cues that underlie changes in competent cells [30]. The establishment of the embryonic root meristem involves the participation of transcription factors that belong to the GRAS family, which includes the SCARECROW (SCR), SCARECROW-LIKE (SCL), and SHORT-ROOT (SHR) proteins, the expressions of which are associated with the distribution of auxins in the root meristem [31]. In the presence of auxins, the expressions of the *PrSCL1* and *CsSCL* genes of *Pinus radiata* and *Castanea sativa* are induced in competent cells at an early stage of root formation [32,33,34]. Additionally, Solé et al. [33] reported *PrSHR* expression in the absence of auxins; therefore, the SCR, SCL, and SHR transcription factors would have a more substantial role before the first cell divisions occur, performing their functions through signaling pathways that are dependent and independent of auxins [35,36]. Considering such assumptions and relying on the spatiotemporal analysis of the morphological modifications linked to the ontogeny of adventitious root formation, we verified an increase in the expressions of the *SCR* and *SHR* genes of *E. nitens* between three and four days after the IBA treatment and before cell division. In this way, we delimited the precise moment of sampling to analyze gene expressions during the induction phase. 

The cutting preparation also affects other regulatory roles that the GRAS proteins perform in multiple biological processes and molecular functions, recently reviewed by Jaiswal and coworkers [37]. The developmental stage interruption of the mother plant is expected to decrease the expression of such transcription factors in the isolated shoot. Two days after hormone treatment, as a result of cell reprogramming, the *SCR* and *SHR* expressions are rearranged in the rooting zone of *E. nitens* shoots, leading to the early molecular events associated with the novo root meristem formation.

### 3.2. Gene Expression Associated with the Induction Phase

#### 3.2.1. Auxin Metabolism

In contemporary studies on model species, researchers have confirmed the central role of auxins in forming adventitious roots. Plants use various mechanisms that control the endogenous auxin levels and modulate their response, including precursors, biosynthesis, transport, and conjugation with other molecules generating different forms of storage [38]. The main route of indoleacetic acid biosynthesis (IAA) uses tryptophan aminotransferase of Arabidopsis1 (TAA1) and the YUCCA enzyme families [39]. Tryptophan is converted to indole-3-pyruvic acid (IPA) by TAA1, and YUCCA converts IPA into IAA. The latter constitutes a limiting step of IAA biosynthesis [40]. Likewise, the active auxin pool can be modulated by releasing precursor and stored forms, such as IBA and IAA conjugates. For a long time, IBA was considered to be a synthetic auxin; however, several researchers have shown that IBA constitutes an endogenous compound, which makes the conversion in IAA necessary to acquire biological activity [41,42,43]. IBA conversion occurs in peroxisomes through a process that is similar to the β-oxidation of fatty acids [44], with a specific transporter (PXA1/CTS/ABCD1) that allows the entry of IBA into the organelle [45]. This pathway is essential for rooting induction in response to IBA treatment. In this regard, Kreiser and coworkers [41], using stable isotope-labeled IBA, internal standards for IAA, and gas chromatography coupled to mass spectrometry, quantified the free IAA form produced by the IBA conversion in the shoots of *Corylus americana* × *C. avellana*, *Ulmus americana*, and *U. pumila* × *U. davidiana*. According to their results, easy-to-root genotypes present high levels of IBA conversion to IAA. We confirmed the leaf expressions of various transcripts encoding enzymes that participate in IBA conversion to IAA, including peroxisomal acyl-CoA dehydrogenase/oxidase (IBR3, T1), enoyl-CoA hydratase (IBR10, T2, T3, and T4), and enoyl-CoA hydratase2 (ECH2, T5). Despite presenting relatively low expression levels, probably associated with the sampling time, their detection reveals the metabolic pathway activity in *E. nitens*.

The endogenous IBA concentration could also be regulated by conjugation and transport mechanisms [46]. Conjugation with aspartic acid and glutamic acid is catalyzed by a group of IAA-amido synthetases that belong to the GRETCHEN HAGEN 3 (GH3) family proteins [47], which play highly redundant roles in the regulation of the endogenous levels [48], presenting activity with IBA and IAA [47]. However, the existence of specific ligase enzymes that catalyze the linking of IBA with amino acids remains unknown. IAA-alanine hydrolase (IAR3) and IAA-amino acid hydrolase (ILL2), which are responsible for breaking the amide bonds, have a greater affinity for those conjugated with IBA, which suggests that their excess could be stored in this chemical form [43]. In response to IBA treatment, we isolated transcripts encoding GH3.1 (T6, T7, and T8) and GH3.5 (T9 and T10) that increased their expressions from one to ten times in the leaves and stem bases. This fact suggests that conjugation with aspartic acid could be the primary regulatory mechanism of the free auxin level. Indeed, IAA (or more likely its amino acid conjugates) oxidation by DIOXYGENASE FOR AUXIN OXIDATION (DAO) family of enzymes and GH3-catalyzed conjugation represents the major contribution to the auxin homeostasis in *Arabidopsis* [48,49]. In agreement with the observations made by Sánchez-García et al. [50], who worked with contrasting carnation cultivars, we did not detect the expressions of transcripts encoding such proteins, which could mean that IAA oxidation is not involved in the main route of IAA homeostasis in *E. nitens*. However, oxidized IAA (oxIAA) can be metabolized by conjugation with glucose, and possibly the glycosylation of IAA to 2-oxindole-3-acetic acid-glucose (IAAox-Glc) by uridine diphosphate glucosyl transferase (UDP glucosyl transferase), decreasing the oxIAA level. We detected the expressions of transcripts encoding UDP-glucosyl transferase 75B1 (T11−T14) and UDP-glucosyl transferase 75B2 (T15 and T16) in leaves and stem bases and a high expression of UDP-glucosyl transferase 74F1 (T17) in the rooting zone. Additionally, we observed the expression of transcripts (T18−T20) encoding hydrolases IAR3 and ILL2 in the leaves, which suggests an IBA regulation by amide conjugation catalyzed by GH3. Additionally, IBA could be conjugated with glucose [51].

The IAA content can also be regulated by the conversion to methyl ester (MeIAA) catalyzed by IAA carboxyl methyltransferase 1 (IAMT1) [52]. We observed a highly significative expression of the *A. thaliana* ortholog *At5g55250* encoding IAMT1 (T21) in both organs. The polar transport regulates the distribution of IAA in a specific direction through the combined action of different transporters [53], in which the adventitious root induction depends, to a large extent, on the auxin metabolism and polar transport [54,55]. The AUXIN-RESISTANT1/LIKE AUX1 (AUX1/LAX) family of transporters acts in the influx of IAA into the cell, while members of the ABCB/PGP family transporters, such as ABCB1, ABCB4, and ABCB9, interact with PIN-FORMED (PIN) family carriers, facilitating their effluxes [56]. We detected a slight increase in the transcript (T22) expression encoding the LAX2 influx carriers in the leaves. Additionally, we observed the increased expressions of transcripts related to the PIN 1 (T23, T24) and PIN7 (T25) efflux proteins in the leaves and stem bases, and we located PIN3 (T26) and PIN6 (T27) in the rooting zones. The subcellular location of the PIN transporters determines the directionality of the IAA flux through the tissues, the functioning and polar movement of which are affected by endogenous and environmental signals that modify the auxin’s distribution [56,57]. 

Similar to IAA, the free and conjugated forms of IBA can be mobilized in plants, the latter being quantitatively the most important form of long-distance transport [58]. Furthermore, the assimilation of free IBA from an exogenous source is a saturable process that supports the hypothesis that the entry of IBA into the cell occurs by the action of specific transporters that are located in the plasma membrane and not by simple diffusion [45]. Likewise, in bioassays with *aux1* mutants, researchers determined that the IBA transport is not altered, confirming that IBA does not constitute a substrate for AUX1. However, other members of the protein family, and in particular LIKE AUX1 (LAX3), in addition to favoring the entry of IAA, would have a discrete affinity for IBA as a substrate [59]. Contrarily, the efflux of IBA is carried out by the action of other transporters than those used by IAA. Indeed, experiments performed with IAA polar transport inhibitors, such as 1-N-naphthylphthalamic acid and 2,3,5-triiodobenzoic acid (TIBA) [57], and the heterologous expressions of PIN2, PIN7, ABCB1, and ABCB19 [60], did not disrupt the IBA movement, which revealed the existence of independent efflux transporters for each molecule. In a recent review of the topic, Damodaran and Strader [61] discussed the IBA transporters identified in *Arabidopsis thaliana*, including PLEIOTROPIC DRUG RESISTANCE (PDR), subfamily G members of the ATP-BINDING CASSETTE (ABCG) transporter family, and TRANSPORTER OF IBA1 (TOB1), and they presupposed other potential IBA transporters that might be involved in plant development. In the case of *E. nitens*, several genes encoding these proteins were expressed in the leaves after the IBA treatment. Simultaneously, we observed a significant increase in the *LAX3* (T28) expression in the rooting zones, which corresponded, in turn, with the increase in the expressions of the genes encoding the ABCG36/ABCG37 (T29−T31) efflux proteins in the leaves. Likewise, we detected a high expression of the *PXA1/ABCD1* gene (T32), which enables the entry of IBA into peroxisomes for its conversion to IAA in the leaf environment.

**Figure 8:**
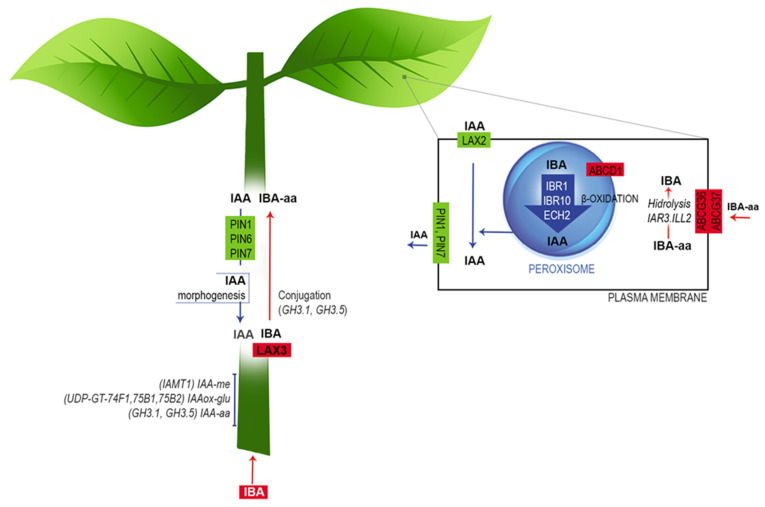
Working model of auxin metabolism during the induction phase of *E. nitens* adventitious root formation. Based on the transcriptome profiles and an extensive literature review, we propose that the free IBA is absorbed by the stem and conjugated with amino acids by the action of LAX3. Next, as an amide-linked conjugated form, IBA is transported to leaves and converted to IAA through β-oxidation. Finally, IAA is transported basipetally via the phloem to the rooting zone, triggering cell dedifferentiation to form root primordium. Further explanations are provided in the text. The figure is adapted from Frick and Strader [43].

In summary, based on the leaf and stem-base transcriptome profiles of *E. nitens* and an extensive literature review, we propose that IBA is absorbed and conjugated with amino acids in the stem to be transported to the leaves, where it is converted to IAA through β-oxidation (Figure 8). Next, the IAA is basipetally moved via the phloem to the rooting zone by the LAX and PIN proteins to promote root initiation. Finally, the IAA excess is controlled by conjugation and methylation.

#### 3.2.2. Hormonal Interaction and Signaling

Root formation constitutes a multiphase development process that begins in response to injuries caused during the cutting preparation, the consequent interruption of water and mineral resources, and the plant signal network [3,62]. Hormones play a critical role in controlling the morphogenic process because they respond to the modification of the environment, provide a signaling network to the organism and are decisive for cell reprogramming. However, the state of knowledge about hormonal homeostasis and the intricate signaling process during the different ontogenic phases is still incipient. 

Cutting separation from the mother plant strongly modifies hormonal homeostasis in the isolated stem. Polar transport plays a leading role in the regulation of the IAA levels and involves the influx (AUX, LAX) and efflux (ABC, PIN, PINOID) transporters considered in the previous section.

At the cellular level, receptor proteins (ABP1; AUXIN BINDING PROTEIN 1) and co-receptors TMK1 (TRANSMEMBRANE KINASE 1) have been proposed to perceive auxins at the cell surface, whereas TIR1/auxin-signaling F box proteins (AFBs) do so in the nucleous [63,64]. 

Once inside the cell, auxins trigger a series of processes that determines the regulation of the target genes [53] involving the Auxin/INDOLE-3-ACETIC ACID (Aux/IAA) and AUXIN RESPONSE FACTOR (ARF) multigenic protein families. The members of both families form dimers with high affinities for one another, and auxin is responsible for regulating these protein interactions in two ways. On the one hand, it positively controls the transcriptions of genes encoding IAA repressor proteins. On the other hand, it promotes the degradation of Aux/IAA repressors through ubiquitination and proteolysis [3]. Under a low auxin concentration, the Aux/IAA repressor forms dimers with ARF proteins and prevents their action as transcriptional factors. However, once IAA increases, it binds to the TIR1/AFB receptors, and together with other proteins, forms a ubiquitination complex (SCFTIR1/AFB) that recognizes and degrades the auxin pathway repressors (Aux/IAA). Once the ARFs are released from the Aux/IAA moderators, they form stable homodimers that activate or repress the expressions of the target genes by binding to their auxin response element (ARE) box, thus enabling interaction with other hormones, such as cytokinins, ethylene, jasmonic acid, and brassinosteroids. During the initiation phase of *E. nitens* root formation, we detected a decrease in the expression of an *A. thaliana* orthologous gene (*at3g62980*) encoding the auxin receptor TIR1 (T33) in leaves, and at the same time, we observed numerous genes that belong to the family of auxin-induced Aux/IAA repressors. Whereas *IAA6* (T34), *IAA13* (T35), and *IAA34* (T36) expressions increased, the *IAA33* (T37) and *AHL20* (T38) expressions were substantially repressed in the leaves, and the transcription of *IAA14* (T39), which is a negative regulator of ARF19, increased more than two-fold in stems, while the mRNAs encoding IAA7 (T40, T41), IAA8 (T42, T43), IAA10 (T44, T45), and ARG4 (T46−T48) were stimulated in both organs.

ARF proteins can be activators and repressors of transcription, depending on the amino acid composition of their domains. For example, ARF6 and ARF8 act as positive regulators, while ARF17 is a negative regulator of adventitious root formation in *A. thaliana* etiolated hypocotyls [65]. We detected the expression of *ARF6* (T49, T50), *ARF8* (T51) and *ARF17* (T52) at the rooting zone of *E. nitens* in vitro shoots, and we observed the transcription of *ARF19* in leaves and stem bases (T53, T54). In conjunction with ARF17, the latter controls the formation of lateral and adventitious roots in *Arabidopsis* [66].

Under daylight conditions, we also observed a significant increase in the *SHY2/IAA3* gene expression (T55, T56) in both organs. *SHY2* (*Short Hypocotyl 2*) encodes IAA3, which is a member of the Aux/IAA family, the expression of which is regulated by light, which suggests a connection between light and the auxin-mediated response [67]. Similarly, the *Small Auxin Up RNAs* (*SAUR*) genes that are present in many plant species are transcriptionally induced by auxins [68]. In *Petunia*, the expressions of the genes encoding SAUR-like proteins are strongly regulated after the cutting excision, with the maximum expression observed between three and six days later, which suggests that their participation during the induction and expression phases is induced by wounding [3]. Likewise, we observed a significant increase in the expression of several transcripts (T57–T66) encoding SAUR-like in the leaves and stem bases during the induction phase (from three to four days after excision) of *E. nitens*. However, researchers have not clarified the biochemical mechanisms explaining how SAUR proteins regulate root development. Similar to Aux/IAA proteins, SAUR activity is controlled by other hormones, including ethylene and jasmonic acid [69].

Cyclins, cyclin-dependent kinases, and their regulatory proteins are involved in auxin- and cytokinin-mediated cell cycle control [70]. Researchers have related the gene expression of cyclins, induced by auxins, to the formation of adventitious roots in *Pinus* and *Quercus* [71,72]. Cyclin B1 and Cyclin D transcriptions increase at the *Petunia* stem base from 2 to 4 days after cutting [73]; Similarly, the expressions of Cyclin D (T67, T68) and the cyclin-dependent kinases CKB1-1 (T69, T70), CKS1 (T71), and CDKA1 (T72) increased in the rooting zone of *E. nitens* over a similar incubation period. Furthermore, Cyclin D is responsible for activating the CYCD−RBR−E2F pathway, which triggers the G1/S phase transition through the association with CDKA [74], which also plays an essential role in the cell cycle progression and resumption in quiescent cells [75]. Likewise, the results obtained by Xu et al. [76], who worked with the transient expression in protoplasts and complementary fluorescence assays, support the fact that *Cyclin D* expression is activated in the initial cortical cells by SHR, SCR, and SCL (T73−T83) determining the root distribution pattern in *Populus*. Based on such experimental evidence, Druege et al. [3] postulated that the expressions of cyclin-encoding genes constitute an important regulatory factor that controls the adventitious rooting exerted by auxins throughout the cell cycle.

Ethylene, the biosynthesis of which is induced by auxins, wounds, and other stresses are considered to be stimulants in the early stage of induction and expression phases, but at the same time, it is also considered an inhibitor in the late stage of induction [62]. While we limited our study to the induction phase, the results are related to those that researchers observed in *Petunia* in the sense that the numerous transcripts encoding the ACC synthase (T84−T91) and ACC oxidase (T92−T100) proteins were stimulated in the leaves and stem bases, which allowed us to suppose a pronounced synthesis of ethylene. Similarly, we observed the expressions of transcripts encoding ethylene receptors (T101−T103) in both organs.

Consequently, many APETALA2/ethylene-responsive element-binding protein (AP2/EREBP) family transcription factors were stimulated in *E. nitens*. Among them, we highlighted the expressions of genes encoding ethylene response proteins (ERFs, T104−T117) because their expressions are promoted by various external stimuli, including abiotic stress [77]. Recently, Zhou et al. [78] demonstrated that ERF109 and ERF115 (T117) stimulate tissue regeneration after damage, and their expressions are controlled by MYC2-mediated jasmonic acid (JA) signaling. 

The other AP2/EREBP family transcription factors that we observed in *E. nitens* include AINTEGUMENTA (ANT, T118), AINTEGUMENTA-like (AIL, T119), and RAV (ABI3/VP1.2, T120). These proteins, together with other members of the subgroup, such as PLETHORA (PLT) and BABY BOOM (BBM), play an essential role in the establishment and maintenance of meristems [78]. Additionally, auxin and ethylene crosstalk are important to the adjustment of several aspects of plant morphogenesis, including root elongation, lateral roots, and trichome formation [79]. IAA promotes ethylene biosynthesis by affecting the ACC synthase gene expression [80], while ethylene regulates the formation of adventitious roots by controlling the localization and transcription of the influx and efflux auxin carriers [8]. 

Similar to ethylene, JA rapidly accumulates at the stem base in response to tissue damage during the cutting preparation [1]. In addition, several authors have correlated the JA peak concentration with adventitious root formation [81,82], confirming that a short JA pulse promotes adventitious rooting in tobacco [83]. The jasmonic acid activity depends on the conjugation with isoleucine and its interaction with F-box proteins [84]. The free JA increase during the adventitious root formation in *A. thaliana* cuttings can be stimulated by the conversion of IBA to AIA, possibly through the overexpressions of the genes involved in nitric oxide-mediated JA biosynthesis [85]. We observed a significant increase (from 2 to 8 times) in the expression of an *Arabidopsis* orthologous gene (*at2g06050*, T121–T127) linked to JA biosynthesis in the leaves and stem bases of *E. nitens*. Additionally, the JA and auxin interaction controls the root primordium formation by expressing genes that belong to the ARF and GH3 protein families that encode the enzymes responsible for the auxin and JA conjugation, determining their activation, inactivation or degradation [86]. Thus, we expected that the interaction of *ARF6* (T49, T50), *ARF8* (T51), and *ARF17* (T52) encoded proteins with those of *GH3.1* (T7, T8), *GH3.3*, *GH3.5* (T9, T10), *GH3.6*, and *GH3.11* would control the balance of the JA conjugation to the active form of jasmonyl-isoleucine against the conjugates of other physiologically inactive amino acids [65].

A low cytokinin/auxin ratio is necessary to stimulate in vitro rooting. Cytokinins antagonistically act with auxins in more than one phase of adventitious rooting and inhibit organogenesis at an early induction stage [87,88]. Experimental data support that cytokinins would inhibit cell differentiation, but do not affect cell proliferation in the root meristem [89] and that zeatin riboside is the main suppressor of the adventitious rooting in *Arabidopsis* hypocotyls [90]. Likewise, cytokinins inhibit root meristem formation by suppressing the genes involved in auxin biosynthesis and transport [91]. In this regard, *Arabidopsis* type-A response regulators, including *AtARR1*, *AtARR10*, and *AtARR12*, directly bind to the promoter region of the *YUC4* gene to block its activation, thereby disrupting auxin synthesis [92]. The inhibitory effect of cytokinins on the adventitious rooting of *Populus trichocarpa* is related to the *PtRR13* (a B-type cytokinin regulator) action on the polar auxin transport [4]. We observed not only a decrease in the gene expression related to the signaling mediated by cytokinins, such as *ARR3* (T128−T130), *ARR9* (T131), and *KNAT3* (T132, T133), but also a marked decrease in the expressions of transcripts that participate in its biosynthesis (T134, T135), and a significant increase in the mRNA encoding the glucosyl transferases (T136−T141) and oxidases (T142, T143) that are involved in the free-form inactivation [93]. These results, as a whole, indicate a regulation at the transcriptomic level that tends, on the one hand, to reduce the expressions of genes related to cytokinin signaling during the induction phase, and on the other hand, to reduce the activity of the hormone through conjugation and oxidation. The alteration in cytokinin homeostasis encourages the auxin/cytokinin ratio that is necessary for the in vitro root formation in *E. nitens* shoots.

There is scarce information in the literature that relates gibberellins (GAs) with the control of the adventitious root formation in woody species. In this regard, Busov et al. [94] showed that the application of GA_3_ inhibits rooting in *Populus* cuttings. They corroborated this negative effect through transgenesis, in which the constitutive overexpression of the *Repressor of GA_1-3_-LIKE 1* or *Giberellic Acid Insensitive* gene increased the number of adventitious roots. Similarly, Mauriat et al. [95] showed that GA_4_ inhibits the root formation in *Arabidopsis* etiolated hypocotyls and *Populus* cuttings; at the same time, the transgenic lines of both species produce fewer roots than the wild type with increased GA biosynthesis or perception. The inhibitory effect of GAs is independent of strigolactone biosynthesis and JA-mediated signaling; however, GAs likely interfere with the establishment of the auxin gradient at the stem base by perturbing the expressions of genes that encode efflux transporters [96]. Additionally, Li et al. [89] observed that the variation in the transcriptomic profile of *Malus* cuttings in response to IBA treatment resulted in a decrease in the expressions of genes associated with GAs synthesis and degradation.

Similarly, in *E. nitens* leaves and stem bases, we observed a reduction in the expressions of transcripts encoding enzymes that are involved in the early stage of biosynthesis (T144−T147) and in those key enzymes that determine the active forms of gibberellins in later stages of the anabolic pathway, including GA_3_ oxidase (T148, T149) and GA_20_ oxidase (T150, T151). At the same time, GA_2_ oxidase (T152) gene transcription substantially increased. The latter catalyzes the conversion of the active GAs, GA_1_, and GA_4_ into their inactive forms, namely GA_8_ and GA_34_ [97]. These results indicate a precise gene regulation of GAs, considering that their signaling process depends on the active-form accumulation mediated by changes in the activities of GA_3_, GA_20_, and GA_2_ oxidases [98]. Furthermore, we highlighted the organ-specific expressions of genes that belong to the *GASA* (*Gibberellic Acid-Stimulated Arabidopsis*) family, and we observed a significant increase (from two to seven times) in the rooting zone (T153, T155−T157), and a decrease in their expressions (from 1.5 to 2 times) in leaves (T154−T156). 

Finally, the interruption of the xylem flow during the cutting preparation triggers a state of water deficit, which increases the ABA level in response to dehydration. ABA inhibits root meristem formation by affecting ethylene and IAA biosynthesis [99], necessitating a high auxin/ABA ratio to promote rhizogenesis [88]. In the analysis of the *E. nitens* transcriptomic variation, we observed, on the one hand, an increase in the *HVA22* gene (T158, T159) that ABA induces in response to dehydration [100], and on the other, a precise regulation that tends to decrease the level of ABA in both organs through the control of the biosynthesis and conjugation. We observed a significant decrease in the expression of the *ABA2* gene (T160), which encodes the alcohol dehydrogenase enzyme that catalyzes the conversion of xanthosine into abscisic aldehyde during ABA biosynthesis, and an increase in transcripts that encode glucosyl transferase (T161−T166), which is responsible for the conjugation of free ABA. 

In summary, the transcriptomic profile variation shows the up-regulation of the gene expressions linked to ethylene and JA biosynthesis and those encoding the GH3 proteins and transcription factors that belong to the AP2/ERBP family. At the same time, it reveals a strong regulation tending toward a decrease in the cytokinin levels, affecting their biosynthesis and conjugation; consequently, it reduces the expressions of the ARRs negative regulators. Likewise, great control over the ABA and GAs gene expression tends to reduce their active forms. This set of actions contributes to the rapid accumulation of IAA in the rooting zone, which is necessary to activate the release of the ARFs proteins from their moderators, AUX/IAA, allowing them to control the expressions of their target genes. This fact will affect the expressions of the genes that encoded cyclins (especially cyclin D), cyclin-dependent kinases, and their regulatory proteins that control the cell cycle activated by SHORT-ROOT, SCARECROW, and SCARECROW-like.

### 3.3. Expressions of Age-Related Genes

The adventitious root formation is regulated by many factors, including genetic, environmental, and developmental elements, which we thoroughly analyzed in the previous section. However, the tree’s chronological age and physiological maturity are the main factors that affect the rooting capacity. In this sense, maturity is a developmental process linked to age, affecting the morphology, growth rate, and other plant physiological traits [101]. The marked decline in the ability to form adventitious roots and develop bud organogenesis and somatic embryos constitutes the most dramatic effect of ageing, seriously limiting the vegetative propagation of adult genotypes.

The mechanisms that underlie the reprogramming of adult cells in the neoformation of organs in forest species are still under study. Elucidating the intrinsic factors of somatic cells that determine their reprogramming to form a root meristem in response to auxinic treatment could be the key factor in reversing the ageing process [2,102]. Greenwood et al. [103] determined that the time required for cell reorganization, the first cell divisions, and cell expansion is similar in the competent hypocotyls and noncompetent epicotyls of *Pinus taeda*. However, the reorientation of the cell division planes that leads to the organization of the root meristem is rarely induced in noncompetent cuttings [35]. According to these results, the auxin priming effect in competent cuttings occurs before cell division resumes. Indeed, the cell division planes reorientation constitutes one of the most evident changes that characterize the initial root cells in competent cuttings compared with the periclinal divisions that arise in stem cells that do not participate in root meristem formation or those cells stimulated by auxins in noncompetent cuttings [10]. In this context, polar transport is required to increase the IAA content at the stem base and its flow and location, determining an asymmetric distribution at the cellular level that will stimulate adventitious rooting [104].

Researchers have associated the recalcitrance phenomenon that they observed in difficult-to-root *Eucalyptus* cuttings with a relatively low auxin content in the cambium section and a high expression of the genes that inhibit the morphogenic process compared to easy-to-root cuttings [22]. Aumond Jr. et al. [23] reported that, in addition to modifying the concentration of IAA, there would be a decrease in the sensitivity to it, which is a fact that would determine the precocity observed in the decline of rooting capacity in *E. globulus* seedlings. Furthermore, the *CsGH3.1* overexpression in *Castanea sativa* microcuttings was negatively correlated with its ability to form roots associated with age, which suggests a leading role for the *CsGH3.1* gene in the regulation of auxin homeostasis in the cells that will originate the root meristem [105]. Similarly, the increase in the IAA concentration or the treatment with urea derivatives improves the auxin transport to the rooting zone, but it does not reverse the recalcitrance in adult *Pinus* cuttings [32], which reveals the possible intervention of other cellular factors. 

In forest species, members of the *Gibberellic Acid Insensitive* (*GAI*), *Repressor of GAI*, and *SCR* (GRAS) gene families are involved in the decline in the rooting capacity that is linked to maturity [32,34,104]. Furthermore, *SCR* and *SCL* were induced in *P. radiata* competent hypocotyls before cell division occurred but were not detected in noncompetent cuttings [104]. In recent years, researchers have made encouraging progress in elucidating the mechanisms that control the cell division plane rotation during root meristem organization [103,106]. Moreover, the mechanical and physical forces that determine the modification in the alignment of microtubules (MTs) are involved in the lateral root formation [107]. Abu-Abied et al. [108] related the cytoskeleton to the decrease in the *E. grandis* rooting capacity through an analysis of auxin-dependent genes that encode several MTs and MT-associated proteins (MAPS), such as TUBULIN, KTANIN, AURORA, MAP65-3, and END BINDING 1b (EB1), in cuttings from juvenile and adult plants. We observed the *AURORA1* (T167) and *MAP65-3* (T168) expressions in the leaves of *E. nitens* juvenile cuttings. Their absence in the rooting zone could be attributed to the sampling time before the first cell division occurred. 

We analyzed the expressions of genes associated with the root meristem determination (*SCR2*, *SCL1* and *AIL1*), auxin biosynthesis, perception, signaling and conjugation (*TAA1*, *TIR1*, *ARF6* and *GH3.1*), and cytokinin signaling (*ARR1*) in shoots harvested from two- to 36-month-old plants. Our results indicate a marked reduction in the rooting capacity concerning the age of the mother plant. The rooting rate decreased from 90% (two-month-old seedlings) to 27 and 13% (24- and 36-month-old plants, respectively). Furthermore, the *SCR2* and *SCL1* gene expression substantially decreased in the stem bases of the 36-month-old shoots. In contrast, the *AIL1* denoted an opposite behavior because its expression was minimal in the juvenile shoots and increased in the 24- and 36-month-old propagules. Regarding the auxin metabolism, the *TAA1* expression (IAA biosynthesis) did not vary, which is attributable to the IBA treatment and its subsequent conversion to IAA; *TIR1* (IAA perception) presented erratic behavior; *ARF6* (IAA signaling) increased due to age effects; while *GH3.1* (IAA conjugation) decreased in adult shoots, and probably as a consequence of a higher free IAA content in the juvenile stage. Interestingly, the *ARR1* expression (cytokinin signaling) increased in the stem bases of the adult shoots. In agreement, higher expression levels of *ARR1* were also associated with age-related loss of rooting capacity in whole microcuttings of *E. globulus* [23].

The quantitative analysis of the selected candidate genes allows us to conclude that the decline in the rooting capacity of *Eucalyptus nitens* is attributable, in part, to the increase in cytokinin signaling (*ARR1*), which negatively affects auxin homeostasis.

## 4. Materials and Methods

### 4.1. Plant Material and Rooting 

For all the experiments, we used Toorongo Plateau provenance *Eucalyptus nitens* (H. Deane and Maiden) Maiden seeds, which we obtained from an orchard established in the Biobio region of Chile. We stored the seeds in a refrigerator at 4 °C until needed. 

Under aseptic conditions, we disinfected the seeds in 70% (*v*/*v*) ethanol for 2 min, transferred them to 1.8% (*w*/*v*) NaOCl and 0.1% Triton X-100 for 20 min, and rinsed them three times with sterile distilled water. Afterwards, we removed the seed coat, and we cultured the zygotic embryos in 11 mL glass tubes (3 seeds/tube) containing 3 mL Murashige and Skoog [109] medium with 0.09 M sucrose (germination medium). We adjusted the pH of the medium to 5.8 before adding 6.5 g/L agar (A-1296, Sigma-Aldrich Inc., St. Louis, MO, USA). We autoclaved the medium at 121 °C and 1.4 kg m^−2^ pressure for 20 min. We incubated the cultures in a growth room at 27 ± 2 °C in the dark for seven days and then placed them under light-emitting diodes (LEDs) mimicking daylight (14 h photoperiod, 116 μmol m^−2^ s^−1^ photosynthetic photon flux density). We either used the seedlings obtained from in vitro seed germination as the source of the explants for the rooting and RNA-Seq experiments, or we transferred to substrate and maintained them under greenhouse conditions until the recalcitrance study. We planted the seedlings in 4 L pots filled with sphagnum peat moss and dolomite (Sun Gro Horticulture Canada LTD., Canada) plus 1.5 g of controlled release micro-fertilize (Osmocote, 18-5-9) and cultured them under partially controlled greenhouse conditions. We kept the plants under 50% natural sunlight radiation and 25–28/18–20 °C (day/night temperature). We kept the substrate water content at field capacity using micro-sprinkler irrigation.

For the RNA-Seq experiment, we rooted the 60-day-old in vitro shoots containing the epicotyl and two phytomers (2–3 cm in length) by pretreatment in a 5 mM indolebutyric acid (IBA, Sigma-Aldrich Inc.) solution for 30 min, before transferring them to the liquid half-strength MS medium lacking plant growth regulators under a temporary immersion system [20].

We rooted shoots harvested from 2-month-old in vitro seedlings for the ageing test using the described procedure. In addition, we superficially disinfected the nodal segments containing two axillary buds collected from the soft branches of 24- and 36-month-old plants by immersion in a 70% ethanol solution for 1 min and transferred them to an aqueous solution of NaOCl (2.2%), to which we added Triton^®^ (0.1%) for 25 min. Finally, we rinsed the explants with sterile distilled water and cultivated them in 45 cc glass tubes containing 10 mL of semisolid MS basal medium (agar 6.5 g/L) with 0.09 M sucrose and subjected them to light conditions. Next, 30-day-old shoots (2–3 cm in length) were separated from the explant and cultivated in a rooting induction medium consisting of half-strength MS semisolid medium (4 g/L Phytagel, Sigma-Aldrich Inc.) with IBA (15 µM) for 96 hs and transferred them to a free-hormone-expression liquid medium. For the temporary immersion program, the shoots were in contact with the medium for 1 min every 4 h. 

We incubated all the cultures under the temperature and light conditions mentioned above.

### 4.2. Tissue Collection

We collected basal stem segments (1 cm) daily from the first to the seventh day following the IBA treatment to determine the ontogenetic phases of the root formation. Next, we harvested the basal stem segments and expanded leaves in the early step (at days three and four) of the induction phase, froze them in liquid nitrogen, and stored them at −80 °C until use for the RNA-Seq experiments. We took three biological replicates each day.

### 4.3. Histological Analysis 

We monitored the cytological changes that occurred at the basal zone of the microshoot by daily sampling, as mentioned in Section 4.2. We fixed the tissue samples in a formalin/ethanol/acetic acid (FAA) solution and dehydrated them with a Biopur^®^ series. We stained the transverse and longitudinal serial sections (10 µm thick) with safranin-Astra blue (Sigma-Aldrich) and mounted them in synthetic Canada balsam (Biopack, Bs. As., Argentina) [110]. We took photomicrographs with a Leica DMLB2 photomicroscope that was equipped with a Leica ICC50HD digital camera (Leica, Wetzlar, Germany).

### 4.4. RNA Isolation, cDNA Synthesis and RNA-Seq Library Preparation

We isolated the total RNA from the stem and leaf samples using the SV Total RNA Isolation System (Promega Corp., Madison, WI, USA) and Total RNA Extraction Kit Spectrum™ Plant (Sigma-Aldrich Inc.), respectively, following the manufacturers’ instructions. We assessed the RNA integrity of all the samples on 2% (m/v) agarose gel stained with ethidium bromide. We determined the quantity by absorption at 260 nm and estimated the purity according to the absorption ratios (260/280 and 260/230, respectively) using a NanoDrop^TM^ 2000 spectrophotometer (Thermo Fisher Scientific, USA). We took three independent biological replicates for further experiments. We synthesized the cDNA used in the qPCR reactions from the high-quality total RNA (5 μg) using the ImProm-II™ reverse transcription system (Promega Corp.) with oligo(dT)20 primers, according to the manufacturers’ instructions. We constructed the libraries used for the RNA-Seq with the TruSeq v2 RNA Sample Preparation Kit (Illumina^®^). We examined the insert sizes with a 2100 Bioanalyzer (Agilent Technologies, Santa Clara, CA, USA) using the High Sensitivity DNA Kit (Agilent Technologies). Finally, we sequenced six cDNA libraries from both organs on an Illumina NextSeq 500 MO platform to produce paired-end reads (2 × 151 bp).

### 4.5. Scarecrow and Short-Root Transcript Fragment Amplifications

We collected basal stem segments (1 cm) from 30 *E. nitens* microshoots for each time point, and we immediately froze them in liquid nitrogen and stored them at –80° C until used for RNA isolation. We extracted the total RNA and synthesized the cDNA as described in Section 4.4. We prepared the RNA from at least three biological replicates. We performed the synthesis of cDNA with 1 µg of total RNA.

We designed several pairs of primers over the conserved regions obtained from an alignment of approximately 40 different nucleotide sequences to obtain the *SCR* and *SHR* sequences with the following parameters: length: 20 pb; fusion temperature: from 59 to 61 °C; GC content: from 55 to 60%; maximum length of amplified fragments: 190 pb (Supplementary Materials, Table S1). 

We individually tested each set of the designed primers with the cDNA synthesized from the basal stem mRNA. We performed the assays as follows: each simplex PCR reaction was composed of 5 μL Green GoTaq^®^ Reaction Buffer 5X (Promega Corp.), 0.5 μL dNTPs 10 mM each, 300 nM of each primer, 0.15 μL GoTaq^®^ DNA Polymerase (5 u/μL), 50 ng cDNA and H_2_O to a final volume of 25 µL. The amplification process consisted of an initial denaturation of 2 min at 95 °C, followed by 35 denaturation cycles at 95 °C for 50 s, 61 °C for 20 s, and 72 °C for 40 s, performing the final extension at 72 °C for 3 min. Finally, we separated the reaction product by electrophoresis on a 1.5% agarose gel in TAE solution and stained it with GelGreen^®^ Nucleic Acid Gel Stain (Biotium, Fremont, CA, USA). After that, we selected the primers SCR_F: TCCAGGGAGATCCGGAATGT and SCR_R: GAACATCCCGAGGAGTAGCG to amplify 159 pb of the SCR gene; and the primers SHR_F: TGCGAGGAGGCGGGTTTAAT and SHR_R: CCCTCCATGCACTAGCCCAA to amplify 168 pb of the SHR gene in the genoma of *E. nitens*. The amplification efficiencies were 92.1 and 95%, respectively.

### 4.6. RNA-Seq Data Processing, De Novo Assembly, and Gene Expression Analysis 

We analyzed the raw-read quality using the FastQC tool (http://www.bioinformatics.babraham.ac.uk/projects/fastqc/, accessed on 3 June 2020). We discarded low-quality reads, and we clipped the adapters used for the library preparation using Trimmomatic v 0.36 [111]. We indexed the *E. grandis* genome (GCF_000612305.1_Egrandis1_0_genomic.fna; available at https://www.ncbi.nlm.nih.gov, accessed on 7 July 2020) with Bowtie2 v2.3.4.1. We use the software Hisat2 v2.1.0 (https://ccb.jhu.edu/software/hisat2/index.shtml, accessed on 8 July 2020) to map the trimmed high-quality reads (both paired-end and single-end reads) on the *E. grandis* genome. Then, we constructed a de novo transcriptome assembly of *E. nitens* using the Trinity v. 2.0.6 package and the genome of *E. grandis* as a guide. We performed the transcriptome assembled validation using the Busco v3 program (https://busco.ezlab.org/, accessed on 4 August 2020). Afterwards, we mapped the trimmed high-quality reads of each sample on the assembled transcriptome using Hisat2 v2.1.0, and the HTSeq-count determined the number of aligned reads that overlapped each transcript. We used the eXpress v1.5.1 (https://pachterlab.github.io/eXpress/overview.html, accessed on 10 August 2020) program for the first estimation of the gene expression. Then, we conducted a differential expression analysis between treatments using the TMM (trimmed mean of M-values) normalization method and negative binomial distribution with default parameters in the Bioconductor package edgeR (https://bioconductor.org/packages/release/bioc/html/edgeR.html, accessed on 12 August 2020). We assigned sequences with fold changes (FC) ≥ 2 in their expressions between treatments, and false discovery rates (FDR) < 0.05 were assigned as differentially expressed transcripts (DETs). Finally, we used BLASTX (https://blast.ncbi.nlm.nih.gov/Blast.cgi, accessed on 13 August 2020) to annotate the assembled transcripts by sequence similarity comparisons. We made comparisons against NCBI nonredundant (nr) and SwissProt protein databases, with an E-value cut-off of 10^−5^. We assigned gene ontology (GO) terms to the transcripts utilizing the BLASTX hits and Blast2Go software with default parameters. We used transDecoder (https://transdecoder.github.io/, accessed on 17 August 2020) software to predict the coding regions in the assembled transcripts, such as the untranslated regions (UTRs), exons, coding sequences (CDs), and mRNAs. Furthermore, we used Trinotate v2.0.2 annotation suite (https://trinotate.github.io/, accessed on 20 August 2020) to identify the protein domains (Pfam database) and to predict the signal peptide sequences (Signal P) and transmembrane domains (TMHMM). Using Trinotate, we also obtained annotations from the EggNOG database. Furthermore, we recognized the up- and down-regulated transcription factors (TFs) by their GO terms or by the BLASTX comparison of the DETs with all the protein sequences of TFs in PlantTFDB 4.0 [112]. Lastly, to assist in the biological interpretation of the changes in the transcriptomic profile, we employed the Mercator pipeline [113] to annotate the DET sequences and MapMan software [114] to represent these changes through colored diagrams of the metabolic pathways.

We described all the transcripts mentioned in the Section 3 in the Supplementary Materials, Table S2, and we represent them in the text as T1, T2, …, Tn.

We randomly selected twelve transcripts, flagged as differentially expressed (up and down) by computational analysis, and we validated them by real-time quantitative PCR (qPCR). We described the RNA isolation and cDNA synthesis in Section 4.4. Each qPCR reaction mixture of 15 µL contained 50 ng cDNA, 7.5 µL 2X SYBR Select Master Mix (Applied Biosystems, Foster City, CA, USA), and 300 nM of the corresponding primer pair. We performed the qPCR reactions in a 7500 Real-Time PCR System (Applied Biosystems) using a program of an initial denaturation at 95 °C for 10 min, and 40 cycles at 95 °C for 15 s and 60 °C for 1 min. We employed the *translation elongation factor 2* (*EF2*) gene as an internal control. We calculated the relative expression value by the delta-delta CT method, and we expressed it as the fold change relative to the expressions in the null controls. We designed the primers used in the qRT-PCR analyses with Primer3 http://primer3plus.com/, accessed on 3 September 2020) y GenScript (www.genscript.com/tools/pcr-primers-designer/standard, accessed on 8 September 2020), (Supplementary Materials, Table S3). We considered three biological replicates for the statistical analysis.

### 4.7. Age-Related Gene Amplification

We selected fourteen age-related genes from the RNA-Seq results and available literature (Supplementary Materials, Table S4). At the induction stage, we assayed the gene expressions in the leaves and basal stems of 2-, 24-, and 36-month-old microshoots by RT-qPCR. We performed the primer design and individual expression quantification in triplicate using the previous reaction conditions.

## Data Availability

We stored the raw sequence in the Sequence Read Archive of The National Center for Biotechnology Information (NCBI), and they are identified with the SRA accession number PRJNA891431.

## Supplementary Materials

The following supporting information can be downloaded at: https://www.mdpi.com/article/10.3390/plants11233301/s1, Figure S1: RNA integrity. Agarose gel electrophoresis of total RNA obtained from stems and leaves extracted before (S0) and after three/four (S3/4) days from IBA treatment; Figure S2: MapMan regulation overview of *E. nitens* leaves (A) and stem bases (B) gene expression during the early step of root formation; Table S1: Gene expression fold changes on day three/four after IBA-treatment, determined by RT-qPCR and RNA-seq analysis; Table S2: Differentially expressed transcripts (DEGs); Table S3: Primers designed for qPCR validation of RNA-seq data; Table S4: Primers designed for the quantification of age-related gene expression.

## References

[1] Steffens B., Rasmussen A. (2016). The physiology of adventitious roots. Plant Physiol..

[2] Díaz-Sala C. (2014). Direct reprogramming of adult somatic cells toward adventitious root formation in forest tree species: The effect of the juvenile—Adult transition. Front. Plant Sci..

[3] Druege U., Franken P., Hajirezaei M.R. (2016). Plant hormone homeostasis, signalling, and function during adventitious root formation in cuttings. Front. Plant Sci..

[4] Pacurar D.I., Perrone I., Bellini C. (2014). Auxin is a central player in the hormone crosstalks that control adventitious rooting. Physiol. Plant..

[5] Xu L. (2018). De novo root regeneration from leaf explants: Wounding, auxin, and cell fate transition. Curr. Opin. Plant Biol..

[6] Mhimdi M., Pérez-Pérez J.M. (2020). Understanding of adventitious root formation: What can we learn from comparative genetics?. Front. Plant Sci..

[7] Druege U., Hilo A., Pérez-Pérez J.M., Klopotek Y., Acosta M., Shahinnia F., Zerche S., Franken P., Hajirezaei M.R. (2019). Molecular and physiological control of adventitious rooting in cuttings: Phytohormone action meets resource allocation. Ann. Bot..

[8] Gonin M., Bergougnoux V., Nguyen T.D., Gantet P., Champion A. (2019). What makes adventitious roots?. Plants.

[9] Li S.-W. (2021). Molecular bases for the regulation of adventitious root generation in plants. Front. Plant Sci..

[10] Pizarro A., Díaz-Sala C. (2019). Cellular dynamics during maturation-related decline of adventitious root formation in forest tree species. Physiol. Plant..

[11] Hamilton M.G., Dutkowski G.W., Joyce K.R., Potts B.M. (2011). Meta-analysis of racial variation in *Eucalyptus nitens* and *E. denticulate*. N. Z. J. For. Sci..

[12] Vega M., Hamilton M.G., Blackburn D.P., McGavin R.L., Baillères H., Potts B.M. (2016). Influence of site, storage and steaming on *Eucalyptus nitens* log-end splitting. Ann. For. Sci..

[13] RIRDC (2009). Trees for farm forestry: 22 promising species. Eucalyptus nitens (Deane and Maiden) Maide. CSIRO For. For. Prod..

[14] Turnbull J.W., Doran J.C., Langkamp P. (1987). Seed development and germination in the Myrtaceae. Germination of Australian Native Plant Seed.

[15] Humara J.M., López M., Casares A., Majada J. (2000). Temperature and provenance as two factors affecting *Eucalyptus nitens* seed germination. Forestry.

[16] Maile N., Nieuwenhuis M. (1996). Vegetative propagation of *Eucalyptus nitens* using stem cuttings. S. Afr. For. J..

[17] Bandyopadhyay S., Cane K., Rasmussen G., Hamill J.D. (1999). Efficient plant regeneration from seedling explants of two commercially important temperate eucalypt species—*Eucalyptus nitens* and *E. globulus*. Plant Sci..

[18] Bandyopadhyay S., Hamill J.D. (2000). Ultrastructural studies of somatic embryos of *Eucalyptus nitens* and comparisons with zygotic embryos found in mature seeds. Ann. Bot..

[19] Gomes F., Canhoto J.M. (2003). Micropropagation of *Eucalyptus nitens* Maiden (shining gum). Vitr. Cell. Dev. Biol. Plant.

[20] Ayala P.G., Brugnoli E.A., Luna C.V., González A.M., Pezzutti R., Sansberro P.A. (2019). *Eucalyptus nitens* plant regeneration from seedling explants through direct adventitious shoot bud formation. Trees.

[21] Di Battista F., Maccario D., Beruto M., Grauso L., Lanzotti V., Curir P., Monroy F. (2019). Metabolic changes associated to the unblocking of adventitious root formation in aged, rooting-recalcitrant cuttings of *Eucalyptus gunnii* Hook. f. (Myrtaceae). Plant Growth Regul..

[22] De Almeida M.R., de Bastiani D., Letaif Gaeta M., de Araújo Mariath J.E., de Costa F., Retallick J., Nolan L., Tai H.H., Strömvik M.V., Fett-Neto A.G. (2015). Comparative transcriptional analysis provides new insights into the molecular basis of adventitious rooting recalcitrance in *Eucalyptus*. Plant Sci..

[23] Aumond M.L., de Araujo A.T., de Oliveira Junkes C.F., de Almeida M.R., Matsuura H.N., de Costa F., Fett-Neto A.G. (2017). Events associated with early age-related decline in adventitious rooting competence of *Eucalyptus globulus* Labill. Front. Plant Sci..

[24] Seppey M., Manni M., Zdobnov E.M. (2019). BUSCO: Assessing genome assembly and annotation completeness. Methods Mol. Biol..

[25] Agulló-Antón M.A., Ferrández Ayela A., Fernández García N., Nicolás C., Albacete A., Pérez Alfocea F., Sánchez Bravo J., Pérez Pérez J.M., Acosta M. (2014). Early steps Nicolás C, of adventitious rooting: Morphology, hormonal profiling and carbohydrate turnover in carnation stem cuttings. Physiol. Plant..

[26] Calderón Baltierra X., Montenegro G., De García E. (2004). Ontogeny of in vitro rooting processes in *Eucalyptus globulus*. Vitr. Cell. Dev. Biol. Plant.

[27] Ballester A., San-José M.C., Vidal N., Fernández-Lorenzo J.L., Vieitez A.M. (1999). Anatomical and biochemical events during in vitro rooting of microcuttings from juvenile and mature phases of chestnut. Ann. Bot..

[28] Naija S., Elloumi N., Jbir N., Ammar S., Kevers C. (2008). Anatomical and biochemical changes during adventitious rooting of apple rootstocks MM 106 cultured in vitro. C. R. Biol..

[29] Porfírio S., Gomes da Silva M.D., Cabrita M.J., Azadi P., Peixe A. (2016). Reviewing current knowledge on olive (*Olea europea* L.) adventitious root formation. Sci. Hortic..

[30] Chupeau M.C., Granier F., Pichon O., Renou J.P., Gaudin V., Chupeau Y. (2013). Characterization of the early events leading to totipotency in an *Arabidopsis* protoplast liquid culture by temporal transcript profiling. Plant Cell.

[31] Jiang K., Feldman L.J. (2005). Regulation of root apical meristem development. Ann. Rev. Cell Dev. Biol..

[32] Sánchez C., Vielba J.M., Ferro E., Covelo G., Solé A., Abarca D., De Mier B.S., Díaz-Sala C. (2007). Two *SCARECROW-LIKE* genes are induced in response to exogenous auxin in rooting-competent cuttings of distantly related forest species. Tree Physiol..

[33] Solé A., Sánchez C., Vielba J.M., Valladares S., Abarca D., Díaz-Sala C. (2008). Characterization and expression of a *Pinus radiata* putative ortholog to the *Arabidopsis*
*SHORT-ROOT* gene. Tree Physiol..

[34] Vielba J.M., Díaz-Sala C., Ferro E., Rico S., Lamprecht M., Abarca D., Ballester A., Sánchez C. (2011). *CsSCL1* is differentially regulated upon maturation in chestnut microshoots and is specifically expressed in rooting-competent cells. Tree Physiol..

[35] Abarca D., Díaz-Sala C. (2009). Reprogramming adult cells during organ regeneration in forest species. Plant Signal. Behav..

[36] Abu-Abied M., Szwerdszarf D., Mordehaev I., Levy A., Belausov E., Yaniv Y., Uliel S., Katzenellenbogen M., Riov J., Ophir R. (2012). Microarray analysis revealed upregulation of nitrate reductase in juvenile cuttings of *Eucalyptus grandis*, which correlated with increased nitric oxide production and adventitious root formation. Plant J..

[37] Jaiswal V., Kakkar M., Kumari P., Zinta G., Gahlaut V., Kumar S. (2022). Multifaceted roles of GRAS transcription factors in growth and stress responses in plants. iScience.

[38] Korasick D.A., Enders T.A., Strader L.C. (2013). Auxin biosynthesis and storage forms. J. Exp. Bot..

[39] Zhao Y., Pua E., Davey M. (2010). The roles of YUCCA genes in local auxin biosynthesis and plant development. Plant Developmental Biology—Biotechnological Perspectives.

[40] Enders T.A., Strader L.C. (2015). Auxin Activity: Past, present, and future. Am. J. Bot..

[41] Kurepin L., Haslam T., Lopez-Villalobos A., Oinam G., Yeung E. (2011). Adventitious root formation in ornamental plants: II. The role of plant growth regulators. Propag. Ornam. Plants.

[42] Kreiser M., Giblin C., Murphy R., Fiesel P., Braun L., Johnson G., Wyse D., Cohen J.D. (2016). Conversion of indole-3-butyric acid to indole-3-acetic acid in shoot tissue of hazelnut (*Corylus*) and elm (*Ulmus*). J. Plant Growth Regul..

[43] Frick E.M., Strader L.C. (2018). Roles for IBA-derived auxin in plant development. J. Exp. Bot..

[44] Epstein E., Ludwig-Müller J. (1993). Indole-3-butyric acid in plants: Occurrence, synthesis, metabolism, and transport. Physiol. Plant..

[45] Strader L.C., Bartel B. (2011). Transport and metabolism of the endogenous auxin precursor indole-3-butyric acid. Mol. Plant.

[46] Ludwig-Müller J. (2011). Auxin conjugates: Their role for plant development and in the evolution of land plants. J. Exp. Bot..

[47] Staswick P.E., Serban B., Rowe M., Tiryaki I., Maldonado M.T., Maldonado M.C., Suza W. (2005). Characterization of an *Arabidopsis* enzyme family that conjugates amino acids to indole-3-acetic acid. Plant Cell.

[48] Hayashi K., Arai K., Aoi Y., Tanaka Y., Hira H., Guo R., Hu Y., Ge C., Zhao Y., Kasahara H. (2021). The main oxidative inactivation pathway of the plant hormone auxin. Nat. Commun..

[49] Mellor N., Band L.R., Pencik A., Novák O., Rashed A., Holman T., Wilson M.H., Voß U., Bishopp A., King J.R. (2016). Dynamic regulation of auxin oxidase and conjugating enzymes AtDAO1 and GH3 modulates auxin homeostasis. Proc. Natl. Acad. Sci. USA.

[50] Sánchez-García A.B., Ibáñez S., Cano A., Acosta M., Pérez-Pérez J.M. (2018). A comprehensive phylogeny of auxin homeostasis genes involved in adventitious root formation in carnation stem cuttings. PLoS ONE.

[51] Zhang G.Z., Jin S.H., Jiang X.Y., Dong R.R., Li P., Li Y.J., Hou B.K. (2016). Ectopic expression of UGT75D1, a glycosyltransferase preferring indole-3-butyric acid, modulates cotyledon development and stress tolerance in seed germination of *Arabidopsis thaliana*. Plant Mol. Biol..

[52] Yang Y., Xu R., Ma C., Vlot A.C., Klessig D.F., Pichersky E. (2008). Inactive methyl indole-3-acetic acid ester can be hydrolyzed and activated by several esterases belonging to the AtMES esterase family of *Arabidopsis*. Plant Physiol..

[53] Matthes M.S., Best N.B., Robil J.M., Malcomber S., Gallavotti A., Mc Steen P. (2019). Auxin EvoDevo: Conservation and diversification of genes regulating auxin biosynthesis, transport, and signaling. Mol. Plant.

[54] Sukumar P., Legué V., Vayssières A., Martin F., Tuskan G.A., Kalluri U.C. (2013). Involvement of auxin pathways in modulating root architecture during beneficial plant-microorganism interactions. Plant Cell Environ..

[55] Luo J., Zhou J.-J., Zhang J.-Z. (2018). Aux/IAA gene family in plants: Molecular structure, regulation, and function. Int. J. Mol. Sci..

[56] Friml J. (2010). Subcellular trafficking of PIN auxin efflux carriers in auxin transport. Eur. J. Cell Biol..

[57] Velada I., Cardoso H., Porfirio S., Peixe A. (2020). Expression profile of PIN-formed auxin efflux carrier genes during IBA-induced in vitro adventitious rooting in *Olea europaea* L.. Plants.

[58] Liu X., Barkawi L., Gardner G., Cohen J.D. (2012). Transport of indole-3-butyric acid and indole-3-acetic acid in *Arabidopsis* hypocotyls using stable isotope labeling. Plant Physiol..

[59] Swarup K., Benkova E., Swarup R., Casimiro I., Peret B., Yang Y., Parry G., Nielsen E., De Smet I., Vanneste S. (2008). The auxin influx carrier LAX3 promotes lateral root emergence. Nat. Cell Biol..

[60] Ruzicka K., Strader L.C., Bailly A., Yang H., Blakeslee J., Langowski L., Nejedlá E., Fujita H., Itoh H., Syōno K. (2010). *Arabidopsis* PIS1 encodes the ABCG37 transporter of auxinic compounds including the auxin precursor indole-3-butyric acid. Proc. Natl. Acad. Sci. USA.

[61] Damodaran S., Strader L.C. (2019). Indole 3-butyric acid metabolism and transport in *Arabidopsis thaliana*. Front. Plant Sci..

[62] Da Costa C., Da T., De Almeida M.R., Ruedell C.M., Schwambach J., Dos Santos Maraschin F., Fett-Neto A.G. (2013). When stress and development go hand in hand: Main hormonal controls of adventitious rooting in cuttings. Front. Plant Sci..

[63] Lakehal A., Chaabouni S., Cavel E., Le Hir R., Ranjan A., Raneshan Z., Novák O., Pacurar D.I., Perrone I., Jobert F. (2019). A molecular framework for the control of adventitious rooting by TIR1/AFB2-Aux/IAA dependent auxin signaling in *Arabidopsis*. Mol. Plant.

[64] Friml J., Gallei M., Gelová Z., Johnson A., Mazur E., Monzer A., Rodriguez L., Roosjen M., Verstraeten I., Živanović B.D. (2022). ABP1–TMK auxin perception for global phosphorylation and auxin canalization. Nature.

[65] Gutiérrez L., Mongelard G., Flokova K., Pacurar D.I., Novak O., Staswick P., Kowalczyk M., Pacurar M., Demailly H., Geiss G. (2012). Auxin controls *Arabidopsis* adventitious root initiation by regulating jasmonic acid homeostasis. Plant Cell.

[66] Li T., González N., Inzé D., Dubois M. (2020). Emerging connections between small RNAs and phytohormones. Trends Plant Sci..

[67] Tian Q., Uhlir N.J., Reed J.W. (2002). *Arabidopsis* SHY2/IAA3 inhibits auxin-regulated gene expression. Plant Cell.

[68] Ren H., Gray W.M. (2015). SAUR proteins as effectors of hormonal and environmental signals in plant growth. Mol. Plant.

[69] Li Z.G., Chen H.W., Li Q.T., Tao J.J., Bian X.H., Ma B., Chen S.-Y., Zhang J.-S. (2015). Three SAUR proteins SAUR76, SAUR77 and SAUR78 promote plant growth in *Arabidopsis*. Sci. Rep..

[70] Komaki S., Sugimoto K. (2012). Control of the plant cell cycle by developmental and environmental cues. Plant Cell Physiol..

[71] Lindroth A.M., Kvarnheden A., Von Arnold S. (2001). Isolation of a PSTAIRE CDC2 cDNA from *Pinus contorta* and its expression during adventitious root development. Plant Physiol. Biochem..

[72] Neves C., Hand P., Amancio S. (2006). Patterns of B-type cyclin gene expression during adventitious rooting of micropropagated cork oak. Plant Cell Tissue Org. Cult..

[73] Ahkami A., Scholz U., Steuernagel B., Strickert M., Haensch K.T., Druege U., Reinhardt D., Nouri E., von Wirén N., Franken P. (2014). Comprehensive transcriptome analysis unravels the existence of crucial genes regulating primary metabolism during adventitious root formation in *Petunia hybrida*. PLoS ONE.

[74] Dewitte W., Scofield S., Alcasada A.A., Maughan S.C., Menges M., Braun N., Collins C., Nieuwland J., Prinsen E., Sundaresan V. (2007). *Arabidopsis* CYCD3 D-type cyclins link cell proliferation and endocycles and are rate-limiting for cytokinin responses. Proc. Natl. Acad. Sci. USA.

[75] Kono A., Umeda-Hara C., Adachi S., Nagata N., Konomi M., Nakagawa T., Uchimiya H., Umeda M. (2007). The *Arabidopsis* D-type cyclin CYCD4 controls cell division in the stomatal lineage of the hypocotyl epidermis. Plant Cell.

[76] Xu M., Liu S., Xuan L., Huang M., Wang Z. (2016). Isolation and characterization of a poplar D-type cyclin gene associated with the SHORT-ROOT/SCARECROW network. Trees.

[77] Licausi F., Ohme-Takagi M., Perata P. (2013). APETALA/Ethylene Responsive Factor (AP2/ERF) transcription factors: Mediators of stress responses and developmental programs. New Phytol..

[78] Zhou W., Lozano-Torres J.L., Blilou I., Zhang X., Zhai Q., Smant G., Li C., Scheres B. (2019). A Jasmonate signaling network activates root stem cells and promotes regeneration. Cell.

[79] Horstman A., Willemsen V., Boutilier K., Heidstra R. (2014). AINTEGUMENTA-LIKE proteins: Hubs in a plethora of networks. Trends Plant Sci..

[80] Zemlyanskaya E.V., Omelyanchuk N.A., Ubogoeva E.V., Mironova V.V. (2018). Deciphering auxin-ethylene crosstalk at a systems level. Int. J. Mol. Sci..

[81] Yamagami T., Tsuchisaka A., Yamada K., Haddon W.F., Harden L.A., Theologis A. (2003). Biochemical diversity among the 1-amino-cyclopropane-1-carboxylate synthase isozymes encoded by the *Arabidopsis* gene family. J. Biol. Chem..

[82] Ahkami A.H., Lischewski S., Haensch K.T., Porfirova S., Hofmann J., Rolletschek H., Melzer M., Franken P., Hause B., Druege U. (2009). Molecular physiology of adventitious root formation in *Petunia hybrida* cuttings: Involvement of wound response and primary metabolism. New Phytol..

[83] Fattorini L., Falasca G., Kevers C., Rocca L.M., Zadra C., Altamura M.M. (2009). Adventitious rooting is enhanced by methyl jasmonate in tobacco thin cell layers. Planta.

[84] Rasmussen A., Hosseini S., Hajirezaei M.-R., Druege U., Geelen D. (2015). Adventitious rooting declines with the vegetative to reproductive switch and involves a changed auxin homeostasis. J. Exp. Bot..

[85] Wasternack C., Song S.S. (2017). Jasmonates: Biosynthesis, metabolism, and signaling by proteins activating and repressing transcription. J. Exp. Bot..

[86] Fattorini L., Veloccia A., Della Rovere F., D’Angeli S., Falasca G., Altamura M.M. (2017). Indole-3-butyric acid promotes adventitious rooting in *Arabidopsis thaliana* thin cell layers by conversion into indole-3-acetic acid and stimulation of anthranilate synthase activity. BMC. Plant Biol..

[87] Chen Q., Westfall C.S., Hicks L.M., Wang S., Jez J.M. (2010). Kinetic basis for the conjugation of auxin by a GH3 family indole-acetic acid-amido synthetase. J. Biol. Chem..

[88] Guan L., Murphy A.S., Peer W.A., Gan L., Li Y., Cheng Z.-M. (2015). Physiological and molecular regulation of adventitious root formation. Crit. Rev. Plant Sci..

[89] Li K., Liang Y., Xing L., Mao J., Liu Z., Dong F., Meng Y., Han M., Zhao C., Bao L. (2018). Transcriptome analysis reveals multiple hormones, wounding and sugar signaling pathways mediate adventitious root formation in apple rootstock. Int. J. Mol. Sci..

[90] Dello Ioio R., Linhares F.S., Scacchi E., Casamitjana-Martinez E., Heidstra R., Costantino P., Sabatini S. (2007). Cytokinins determine Arabidopsis root-meristem size by controlling cell differentiation. Curr. Biol..

[91] Kuroha T., Satoh S. (2007). Involvement of cytokinins in adventitious and lateral root formation. Plant Root.

[92] Mao J., Zhang D., Meng Y., Li K., Wang H., Han M. (2019). Inhibition of adventitious root development in apple rootstocks by cytokinin is based on its suppression of adventitious root primordia formation. Physiol. Plant..

[93] Kieber J.J., Schaller G.E. (2018). Cytokinin signaling in plant development. Development.

[94] Busov V., Meilan R., Pearce D.W., Rood S.B., Ma C., Tschaplinski T.J., Strauss S.H. (2006). Transgenic modification of gai or rgl1 causes dwarfing and alters gibberellins, root growth, and metabolite profiles in *Populus*. Planta.

[95] Mauriat M., Petterle A., Bellini C., Moritz T. (2014). Gibberellins inhibit adventitious rooting in hybrid aspen and *Arabidopsis* by affecting auxin transport. Plant J..

[96] Lakehal A., Bellini C. (2019). Control of adventitious root formation: Insights into synergistic and antagonistic hormonal interactions. Physiol. Plant..

[97] Hedden P., Proebsting W.M. (1999). Genetic analysis of gibberellin biosynthesis. Plant Physiol..

[98] Hauvermale A.L., Ariizumi T., Steber C.M. (2012). Gibberellin signaling: A theme and variations on DELLA repression. Plant Physiol..

[99] McAdam S.A.M., Brodribb T.J., Ross J.J. (2016). Shoot-derived abscisic acid promotes root growth. Plant Cell Environ..

[100] Gomes Ferreira M.D., Araújo Castro J., Santana Silva R.J., Michelia F. (2019). HVA22 from Citrus: A small gene family whose some members are involved in plant response to abiotic stress. Plant Physiol. Biochem..

[101] Day M., Greenwood M.S., Díaz-Sala C. (2002). Age and size-related trends in woody plant shoot development: Regulatory pathways. Tree Physiol..

[102] Díaz-Sala C., Park Y.-S., Bonga J.M., Moon H.-K. (2016). Physiological, cellular, molecular and genomic analysis of the effect of maturation on propagation capacity. Vegetative Propagation of Forest Trees.

[103] Greenwood M.S., Cui X., Xu F. (2001). Response to auxin changes during maturation-related loss of adventitious rooting competence in loblolly pine (*Pinus taeda*) stem cuttings. Physiol. Plant..

[104] Abarca D., Pizarro A., Hernández I., Sánchez C., Solana S.P., del Amo A., Carneros E., Díaz-Sala C. (2014). The GRAS gene family in pine: Transcript expression patterns associated with the maturation-related decline of competence to form adventitious roots. BMC Plant Biol..

[105] Vielba J.M., Varas E., Rico S., Covelo P., Sánchez C. (2016). Auxin-mediated expression of a *GH3* gene in relation to ontogenic state in chestnut. Trees.

[106] Pizarro A., Díaz-Sala C. (2022). Expression levels of genes encoding proteins involved in the cell wall–plasma membrane–cytoskeleton continuum are associated with the maturation-related adventitious rooting competence of pine stem cuttings. Front. Plant Sci..

[107] Vilches-Barro A., Maizel A. (2015). Talking through walls: Mechanisms of lateral root emergence in *Arabidopsis thaliana*. Curr. Opin. Plant Biol..

[108] Abu-Abied M., Szwerdszarf D., Mordehaev I., Yaniv Y., Levinkron S., Rubinstein M., Riov J., Ophir R., Sadot E. (2014). Gene expression profiling in juvenile and mature cuttings of *Eucalyptus grandis* reveals the importance of microtubule remodeling during adventitious root formation. BMC Genom..

[109] Murashige T., Skoog F. (1962). A revised medium for rapid growth and bioassays with tobacco tissue cultures. Physiol. Plant..

[110] González A.M., Cristóbal C.L. (1997). Anatomía y ontogenia de semillas de *Helicteres lhotzkyana* (Sterculiaceae). Bonplandia.

[111] Bolger A.M., Lohse M., Usadel B. (2014). Trimmomatic: A flexible trimmer for Illumina sequence data. Bioinformatics.

[112] Jin J., Tian F., Yang D.C., Meng Y.Q., Kong L., Luo J., Gao G. (2017). PlantTFDB 4.0: Toward a central hub for transcription factors and regulatory interactions in plants. Nucleic Acids Res..

[113] Lohse M., Nagel A., Herter T., May P., Schroda M., Zrenner R., Tohge T., Fernie A.R., Stitt M., Usadel B. (2014). Mercator: A fast and simple web server for genome scale functional annotation of plant sequence data. Plant. Cell Environ..

[114] Thimm O., Bläsing O., Gibon Y., Nagel A., Meyer S., Krüger P., Stitt M. (2004). MAPMAN: A user-driven tool to display genomics data sets onto diagrams of metabolic pathways and other biological processes. Plant J..

