# Supplementary material for: Transcriptome Dynamics of Rooting Zone and Leaves during In Vitro Adventitious Root Formation in Eucalyptus nitens
Source: Plants (Basel). 2022 Nov 29;11(23):3301. doi: 10.3390/plants11233301 (PMC9740172; doi:10.3390/plants11233301)
Supplement: Supplementary file 1 [file plants-11-03301-s001.zip › plants-2013263-supplementary.pdf]

Supplementary material

Article

Transcriptome Dynamics of Rooting Zone and Leaves During in vitro Adventitious Root Formation in *Eucalyptus nitens*

Paula Ayala, Raúl Acevedo, Claudia Luna, Máximo Rivarola, Cintia Acuña, Susana Marcucci Poltri, Ana González and Pedro Sansberro

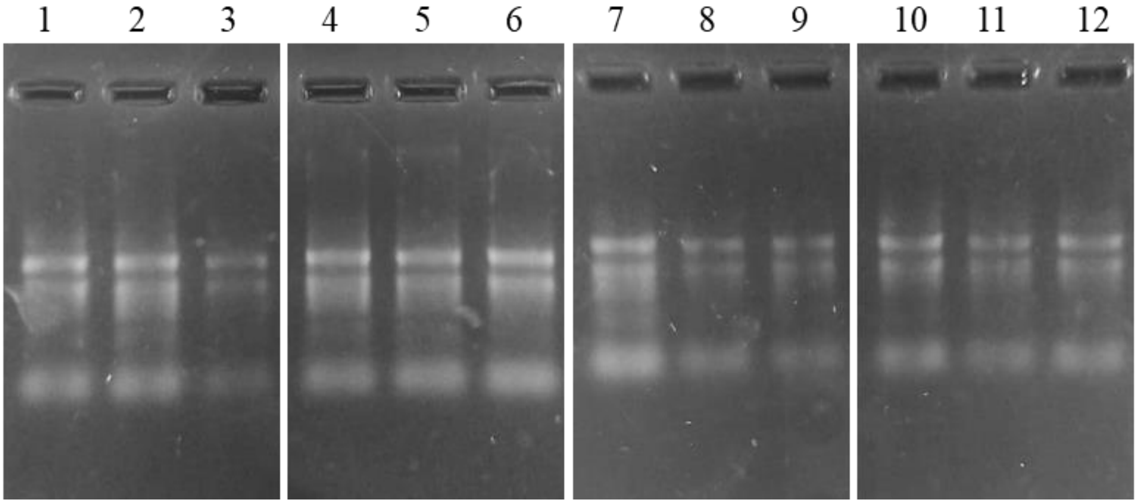

Figure S1. RNA integrity. Agarose gel electrophoresis of total RNA obtained from stems and leaves extracted before (S0) and after three/four (S3/4) days from IBA treatment. Three biological replicates per sample were used. Stem bases: S0, lanes 1, 2 and 3; S3/4, lanes 4, 5 and 6. Leaves: S0; lanes 7, 8 and 9; S3/4, lanes 10, 11 and 12).

RNA integrity was analyzed by horizontal 2% agarose gel electrophoresis and stained with GelGreen®. Total RNA concentrations varied between 436.4 and 1,301.1 ng/µL. The purity ratio A260/280 varied between 1.89 and 2.05, while A260/230 ranged between 1.86 and 2.08. No RNA degradation was observed.

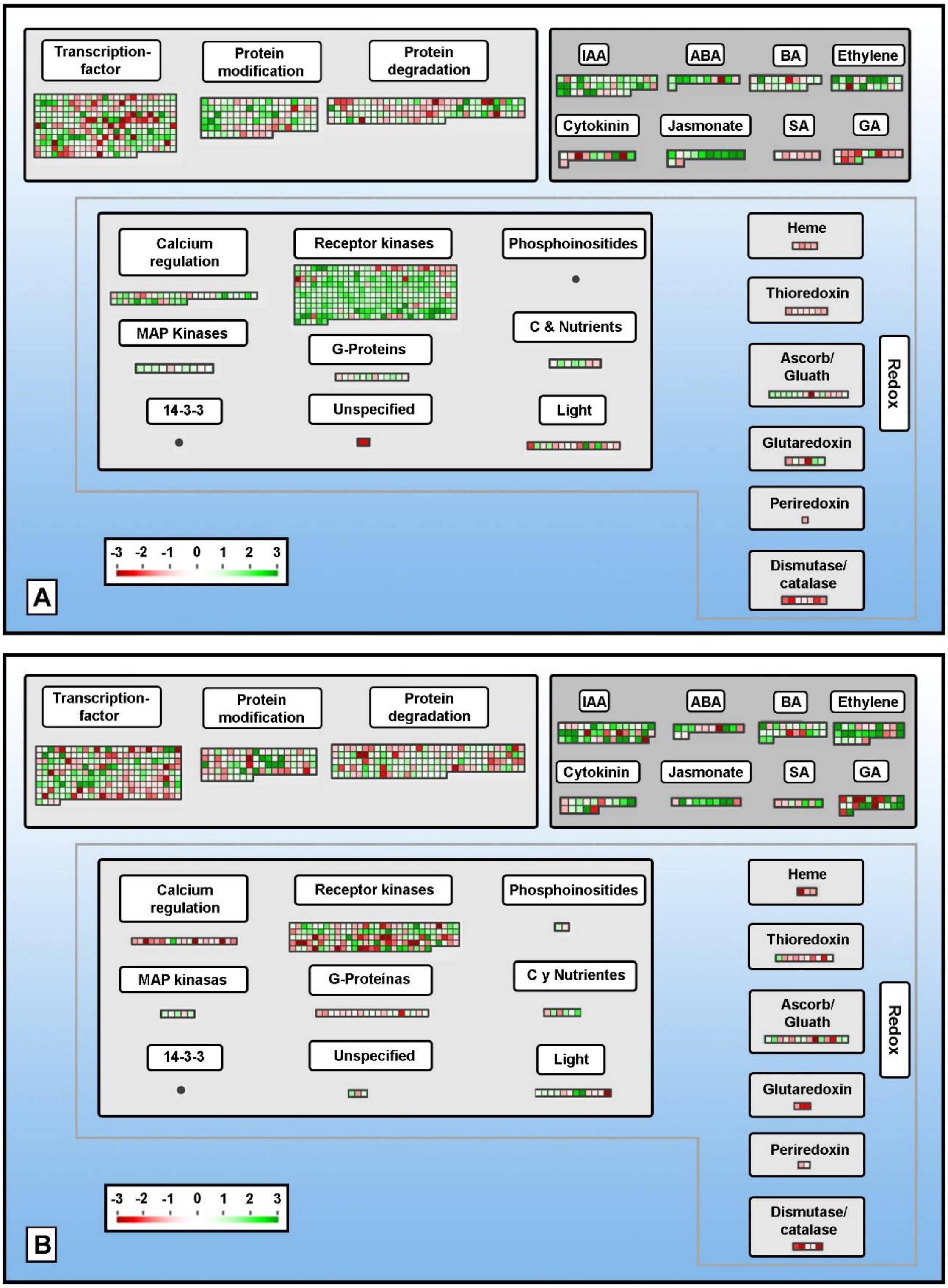

Figure S2. MapMan regulation overview of *E. nitens* leaves (**A**) and stem bases (**B**) gene expression during the early step of root formation. Each square corresponds to a gene. Red and green indicate lower and higher expression than the control, respectively. The scale bar is shown in log2. Sequences with a fold change (FC)≥2 in its expression between treatments and FDR<0.05 were assigned as a differentially expressed transcript.

Table S1. Gene expression fold changes on day three/four after the IBA treatment, determined by RT-qPCR and RNA-seq analysis.

**Gene Name**	**qPCR**	**ARN-seq**
Endoglucanase 1 (Endo1)	6,25	6,29
Citokinin dehydrogenase (CitokDes3)	4,75	5,36
Ethylene-responsive transcription tactor (ERF003)	2,46	4,46
1-aminocyclopropane-1-carboxylate oxidase (ACO)	-2,83	-1,81
Fructose-bisphosphate aldolase (FBA)	-4,64	-2,02
Translation elongation factor 2 (EF 2)	1	1
Isoprene synthase, chloroplastic-like (ISC-like)	-6,64	-5,18
Gretchen hagen 3 (GH3-1)	3,54	8,42
Cellulose synthase A catalytic 8 (CSACat8)	-4,05	-3,04
Auxin response factors (ARF6)	0,23	1,03
Scarecrow (SCR)	1,5	1,93

Table S2. Differentially expressed transcripts (DETs).

Sigla	Transcript	Annotation	Function	Log_2 FC
Auxins metabolisms				
T1	tr3710_c79_g1	Acyl-CoA dehydrogenase/oxidase (IBR3)	IBA to IAA conversion	ns L
T2	tr1960_c212_g1	Enoyl-CoA hydratase (IBR10)	IBA to IAA conversion	↑ 1.0 L
T3	tr905_c81_g1	Enoyl-CoA hydratase (IBR10)	IBA to IAA conversion	↑ 1.1 L
T4	tr1960_c199_g2	Enoyl-CoA hydratase (IBR10)	IBA to IAA conversion	↑ 0.7 L
T5	tr5933_c23_g1	Enoyl-CoA hydratase 2 (ECH2)	IBA to IAA conversion	ns L
T6	tr496_c723_g1	GRETCHEN HAGEN 3<br>IAA-amida synthase GH3.1	IAA - amino acid conjugation	↑ 3.8 L<br>↑ 2.9 S
T7	tr1863_c28_g12	GRETCHEN HAGEN 3<br>IAA-amida synthase GH3.1	IAA - amino acid conjugation	↑ 7.2 L<br>↑ 8.4 S
T8	tr1863_c28_g8	GRETCHEN HAGEN 3<br>IAA-amida synthase GH3.1	IAA - amino acid conjugation	↑ 9.6 L<br>↑ 10.3 S
T9	tr4738_c576_g1	GRETCHEN HAGEN 3<br>IAA-amida synthase GH3.5	IAA - amino acid conjugation	↑ 1.1 L<br>↑ 1.0 S
T10	tr6569_c202_g1	GRETCHEN HAGEN 3<br>IAA-amida synthase GH3.5	IAA - amino acid conjugation	↑ 1.1 L<br>↑ 2.8 S
T11	tr1921_c236_g3	Uridine 5'-diphosphate-glucose:indol-3-ylacetyl)-beta-D-glucosyl transferase<br>UDP-glucosyltransferase	IAA - sugar conjugation	↑ 0.8 L
T12	tr1921_c245_g1	Uridine 5'-diphosphate-glucose:indol-3-ylacetyl)-beta-D-glucosyl transferase<br>UDP-glucosyltransferase	IAA - sugar conjugation	↑ 0.7 L
T13	tr268_c28_g1	Uridine 5'-diphosphate-glucose:indol-3-ylacetyl)-beta-D-glucosyl transferase<br>UDP-glucosyltransferase 75B1	IAA - sugar conjugation	↑ 0.8 L<br>↑ 1.1 S
T14	tr1939_c3_g1	Uridine 5'-diphosphate-glucose:indol-3-ylacetyl)-beta-D-glucosyl transferase<br>UDP-glucosyltransferase	IAA - sugar conjugation	↑ 0.7 L
T15	tr1921_c236_g9	Uridine 5'-diphosphate-glucose:indol-3-acetyl)-beta-D-glucosyl transferase<br>UDP-glucosyltransferase	IAA - sugar conjugation	↑ 1.4 L
T16	tr268_c33_g3	Uridine 5'-diphosphate-glucose:indol-3-acetyl)-beta-D-glucosyl transferase<br>UDP-glucosyltransferase 75B2	IAA - sugar conjugation	↑ 1.5 L<br>↑ 1.7 S
T17	tr3678_c14_g2	Uridine 5'-diphosphate-glucose:indol-3-acetyl)-beta-D-glucosyl transferase<br>UDP-glucosyltransferase 74F1	IAA - sugar conjugation	↑ 2.3 S
T18	tr5846_c882_g1	IAR3/ILL2 hydrolase	Amide-bond cleavage	↑ 0.8 S
T19	tr4275_c68_g2	IAR3/ILL2 hydrolase	Amide-bond cleavage	↑ 0.9 S
T20	tr4002_c17_g2	IAR3/ILL2 hydrolase	Amide-bond cleavage	↑ 0.9 S
T21	tr5825_c150_g1	IAA carboxyl methyltransferase 1 (IAMT1).	IAA methylation	↑ 4.1 L<br>↑ 6.4 S
T22	tr2658_c159_g1	LIKE AUXIN RESISTANT2 (LAX2)	IAA influx carrier	↑ 1.6 L
T23	tr7297_c420_g1	PIN1	IAA efflux carrier	↑ 1.8 L<br>↑ 1.7 S
T24	tr3578_c13_g1	PIN1	IAA efflux carrier	↑ 1.1 L
T25	tr5015_c923_g1	PIN7	IAA efflux carrier	↑ 1.2 L<br>↑ 1.4 S
T26	tr2528_c174_g1	PIN3	IAA efflux carrier	↑ 1.0 S
T27	tr5878_c121_g1	PIN6	IAA efflux carrier	↑ 1.9 S
T28	tr2658_c159_g1	LIKE AUXIN RESISTANT3 (LAX3)	IAA/IBA influx carrier	↑ 2.8 S
T29	tr4959_c346_g1	ABCG36/ABCG37	IBA efflux carrier	↑ 3.1 L
T30	tr908_c134_g1	ABCG36/ABCG37	IBA efflux carrier	↑ 3:1 L
T31	tr7363_c12_g1	ABCG36/ABCG37	IBA efflux carrier	↑ 3.1 L

T32	tr5920_c209_g1	PXA1/ABCD1	IBA efflux carrier	↑ 2.2 H
Hormonal interactions and signaling				
T33	tr7325_c340_g1	Transport Inhibitor Response 1 (TIR1)	Auxin receptor	↓-1.4 L
T34	tr5854_c681_g1	IAA6 transcription factor	Aux/IAA protein family gene	↑ 2.1 L
T35	tr2018_c21_g1	IAA13 transcription factor	Aux/IAA protein family gene	↑ 1.3 L
T36	tr3553_c114_g1	IAA34 transcription factor	Aux/IAA protein family gene	↑ 2.0 L
T37	tr3909_c8_g1	IAA33 transcription factor	Aux/IAA protein family gene	↓-4.4 L
T38	tr2100_c21_g1	AHL20 transcription factor	Aux/IAA protein family gene	↓-2.1 L
T39	tr4032_c79_g1	IAA14 transcription factor	Aux/IAA protein family gene	↑ 2.9 S
T40	tr937_c94_g1	IAA7 transcription factor	Aux/IAA protein family gene	↑ 2.4 L<br>↑ 1.0 S
T41	tr1963_c18_g1	IAA7 transcription factor	Aux/IAA protein family gene	↑ 1.3 S
T42	Tr1925_c27_g1	IAA8 transcription factor	Aux/IAA protein family gene	↑ 1.0 L<br>↑ 1.6 S
T43	tr5955_c127_g2	IAA8 transcription factor	Aux/IAA protein family gene	↑ 0.6 L
T44	3866_c90_g2	IAA10 transcription factor	Aux/IAA protein family gene	↑ 2.3 L<br>↑ 3.1 S
T45	tr1260_c1_g1	IAA10 transcription factor	Aux/IAA protein family gene	↑ 2.4 L<br>↑ 4.7 S
T46	tr1960_c281_g1	ARG4 transcription factor	Aux/IAA protein family gene	↑ 1.5 L<br>↑ 1.7 S
T47	tr1963_c17_g10	ARG4 transcription factor	Aux/IAA protein family gene	↑ 1.4 L<br>↑ 1.6 S
T48	tr1963_c17_g8	ARG4 transcription factor	Aux/IAA protein family gene	↑ 1.3 L<br>↑ 1.6 S
T49	tr4785_c211_g1.4	Auxin Response Factor ARF6	Mediate auxin responses	↑ 1.2 S
T50	tr5939_c74_g2	Auxin Response Factor ARF6	Mediate auxin responses	↑ 0.9 S
T51	tr5015_c534_g1	Auxin Response Factor ARF8	Mediate auxin responses	↑ 1.2 S
T52	tr5921_c82_g1	Auxin Response Factor ARF17	Mediate auxin responses	ns S
T53	tr4280_c47_g1	Auxin Response Factor ARF19	Mediate auxin responses	↑ 1.1 L<br>↑ 1.0 S
T54	tr5939_c74_g2	Auxin Response Factor ARF19	Mediate auxin responses	↑ 0.9 S
T55	Tr1963_c17_g6	SHY2/IAA3 transcription factor	Aux/IAA protein family gene	↑ 1.4 L<br>↑ 2.1 S
T56	tr7325_c339_g1	SHY2/IAA3 transcription factor	Aux/IAA protein family gene	↑ 1.9 L<br>↑ 5.5 S
T57	tr496_c1164_g2	Small Auxin Up RNAs. SAUR-like (SAUR68)	Auxin responsive genes. SAUR family	↑ 1.7 L<br>↑ 3.8 S
T58	tr496_c909_g3	Small Auxin Up RNAs. SAUR-like	Auxin responsive genes. SAUR family	↑ 1.0 L<br>↑ 1.4 S
T59	tr5943_c263_g2	Small Auxin Up RNAs. SAUR-like	Auxin responsive genes. SAUR family	↑ 1.7 L
T60	tr496_c1254_g9	Small Auxin Up RNAs. SAUR-like	Auxin responsive genes. SAUR family	↑ 3.0 S
T61	tr496_c1254_g7	Small Auxin Up RNAs. SAUR-like	Auxin responsive genes. SAUR family	↑ 3.1 S
T62	tr496_c1254_g10	Small Auxin Up RNAs. SAUR-like	Auxin responsive genes. SAUR family	↑ 2.1 S
T63	tr496_c750_g1	Small Auxin Up RNAs. SAUR-like	Auxin responsive genes. SAUR family	↑ 2.1 S
T64	tr5293_c0_g1	Small Auxin Up RNAs. SAUR-like	Auxin responsive genes. SAUR family	↑ 6.3 S
T65	tr496_c558_g2	Small Auxin Up RNAs. SAUR-like	Auxin responsive genes. SAUR family	↑ 2.2 S
T66	tr2672_c0_g1	Small Auxin Up RNAs. SAUR-like	Auxin responsive genes. SAUR family	↑ 1.2 S
T67	tr5839_c72_g1	Cyclin D-type (CCD3-1)	Cell cycle control	↑ 1.1 S

T68	tr530_c92_g1	Cyclin D-type (CCD3-1)	Cell cycle control	↑ 1.0 S
T69	tr5934_c173_g1	Cyclin-dependent kinase 1 (CKB1-1)	Cell cycle control	↑ 1.6 S
T70	tr4991_c487_g1	Cyclin-dependent kinase 1 (CKB1-1)	Cell cycle control	↑ 1.4 S
T71	tr931_c33_g1	Cyclin-dependent kinase 1 (CKS1)	Cell cycle control	ns S
T72	tr2103_c159_g1	Cyclin-dependent kinase 1 (CDKA1)	Cell cycle control	↑ 0.6 S
T73	tr5864_c693_g1	SCARECROW-like 5 (SCL5)	Transcriptional regulator. GRAS family	↑ 1.3 S
T74	tr6540_c351_g1	SCARECROW-like 13 (SCL13)	Transcriptional regulator. GRAS family	↓-0,7 L
T75	tr7260_c339_g1	SCARECROW-like 13 (SCL13)	Transcriptional regulator. GRAS family	↓-0,9 S
T76	tr3686_c605_g1	SCARECROW-like 14 (SCL14)	Regulador transcripcional familia GRAS	↑ 1.2 L<br>↑ 1.9 S
T77	tr972_c169_g3	SCARECROW-like 14 (SCL14)	Transcriptional regulator. GRAS family	↑ 0.9 L
T78	tr3686_c713_g1	SCARECROW-like 14 (SCL14)	Transcriptional regulator. GRAS family	↑ 0.6 L
T79	tr3686_c530_g1	SCARECROW-like 14 (SCL14)	Transcriptional regulator. GRAS family	↑ 0.9 L<br>↑ 1.1 S
T80	tr3686_c710_g1	SCARECROW-like 14 (SCL14)	Transcriptional regulator. GRAS family	↑ 1.3 L
T81	tr3686_c814_g5	SCARECROW-like 14 (SCL14)	Transcriptional regulator. GRAS family	↑ 0.8 S
T82	tr5916_c1149_g3	SCARECROW-like 21 (SCL21)	Transcriptional regulator. GRAS family	↑ 1.3 L
T83	tr4988_c126_g1	SCARECROW-like 21 (SCL21)	Transcriptional regulator. GRAS family	↑ 2.0 S
T84	tr4752_c258_g4	1-aminocyclopropane-1-carboxylate synthase 1 (ACC1)	Biosíntesis de etileno	↑ 1.1 L<br>↑ 2.5 S
T85	tr43_c3_g1	1-aminocyclopropane-1-carboxylate synthase 1 (ACC1)	Ethylene biosynthesis	↑ 1.2 L<br>↑ 2.7 S
T86	tr3461_c0_g2	1-aminocyclopropane-1-carboxylate synthase 1 (ACC1)	Ethylene biosynthesis	↑ 4.5 S
T87	tr6569_c89_g1	1-aminocyclopropane-1-carboxylate synthase 2 (ACC2)	Ethylene biosynthesis	↑ 3.1 L<br>↑ 4.4 S
T88	tr2886_c3_g2	1-aminocyclopropane-1-carboxylate synthase 2 (ACC2)	Ethylene biosynthesis	↑ 2.0 S
T89	tr4752_c258_g3	1-aminocyclopropane-1-carboxylate synthase 2 (ACC2)	Ethylene biosynthesis	↑ 2.1 S
T90	tr5955_c244_g1	1-aminocyclopropane-1-carboxylate synthase 4 (ACC4)	Ethylene biosynthesis	↑ 6.2 L<br>↑ 3.4 S
T91	tr1877_c23_g1	1-aminocyclopropane-1-carboxylate synthase 4 (ACC4)	Ethylene biosynthesis	↑ 4.6 L<br>↑ 3.2 S
T92	tr5020_c252_g1	1-aminocyclopropane-1-carboxylate oxidase (ACC oxidase)	Ethylene biosynthesis	↑ 1.4 L<br>↑ 1.9 S
T93	tr3855_c80_g1	1-aminocyclopropane-1-carboxylate oxidase (ACC oxidase)	Ethylene biosynthesis	↑ 6.3 L<br>↑ 5.7 S
T94	Tr1921_c200_g1	1-aminocyclopropane-1-carboxylate oxidase (ACC oxidase)	Ethylene biosynthesis	↑ 9.8 L<br>↑ 6.1 S
T95	tr4834_c160_g1	1-aminocyclopropane-1-carboxylate oxidase (ACC oxidase)	Ethylene biosynthesis	↑ 1.2 L
T96	Tr5534_c0_g1	1-aminocyclopropane-1-carboxylate oxidase (ACC oxidase)	Ethylene biosynthesis	↑ 1.1 L
T97	tr7260_c403_g2	1-aminocyclopropane-1-carboxylate oxidase (ACC oxidase)	Ethylene biosynthesis	↑ 1.2 L
T98	tr4269_c56_g1	1-aminocyclopropane-1-carboxylate oxidase (ACC oxidase)	Ethylene biosynthesis	↓-3.6 L

T99	tr7299_c221_g2	1-aminocyclopropane-1-carboxylate oxidase (ACC oxidase)	Ethylene biosynthesis	↑ 0.9 S
T100	tr4991_c501_g1	1-aminocyclopropane-1-carboxylate oxidase (ACC oxidase)	Ethylene biosynthesis	↓-1.5 S
T101	tr7325_c185_g1	Ethylene receptor subfamily 1 (ERS1)	Ethylene receptor	↑ 0.8 S
T102	tr2039_c114_g1	Ethylene receptor, subfamily 2 (ERS2)	Ethylene receptor	↓-0.7 L
T103	tr2100_c61_g1	Ethylene response 2 (ETR2)	Ethylene receptor	↑ 1.2 S
T104	tr566_c73_g1	Ethylene Response Factor. ABA REPRESSOR1 (ABR1)	Transcriptional regulator. AP2/EREBP family	↑ 1.3 L
T105	tr3742_c81_g1	Ethylene Response Factor. (ERF)	Transcriptional regulator. AP2/EREBP family	↑ 0.7 L
T106	tr5875_c146_g1	Ethylene Response Factor. (ERF)	Transcriptional regulator. AP2/EREBP family	↑ 3.0 L<br>↑ 4.5 S
T107	tr566_c73_g1	Ethylene Response Factor. (ERF)	Transcriptional regulator. AP2/EREBP family	↑ 0.9 S
T108	tr2674_c89_g1	Ethylene Response Factor 1 (ERF1)	Transcriptional regulator. AP2/EREBP family	↑ 1.3 S
T109	tr3743_c922_g1	Ethylene Response Factor 1 (ERF1)	Transcriptional regulator. AP2/EREBP family	↑ 1.2 S
T110	tr3961_c11_g1	Ethylene Response Factor 1 (ERF1)	Transcriptional regulator. AP2/EREBP family	↓-2.8 S
T111	tr7299_c142_g1	Ethylene Response Factor 1 (ERF1)	Transcriptional regulator. AP2/EREBP family	↑ 1.4 L
T112	tr6442_c0_g1	Ethylene Response Factor 1 (ERF1)	Transcriptional regulator. AP2/EREBP family	↑ 1.6 L
T113	tr3743_c922_g1	Ethylene Response Factor 4 (ERF4)	Transcriptional regulator. AP2/EREBP family	↑ 1.1 L
T114	tr7297_c516_g1	Ethylene Response Factor 110 (ERF110)	Transcriptional regulator. AP2/EREBP family	↑ 1.6 L<br>↑ 3.5 S
T115	tr5854_c451_g1	Ethylene Response Factor 110 (ERF110)	Transcriptional regulator. AP2/EREBP family	↑ 1.2 L
T116	tr7316_c200_g1	Ethylene Response Factor 110 (ERF110)	Transcriptional regulator. AP2/EREBP family	↑ 1.9 S
T117	tr3743_c922_g1	Ethylene Response Factor 115 (ERF115)	Transcriptional regulator. AP2/EREBP family	↑ 1.1 L<br>↑ 1.2 S
T118	tr1877_c153_g9	AINTEGUMENTA (ANT)	Transcriptional regulator. AP2/EREBP family	↑ 1.6 L
T119	tr531_c93_g1	AINTEGUMENTA-like 6 (AIL6)	Transcriptional regulator. AP2/EREBP family	↑ 3.0 L<br>↑ 3.4 S
T120	tr3746_c101_g1	Factor de transcripción RAV (ABI3/VP1 2)	Transcriptional regulator. AP2/EREBP family	↑ 0.7 L
T121	tr4885_c0_g1	12-oxophytodienoate reductase 2 (12-Oxo-PDA-reductase)	Jasmonic acid biosynthesis	↑ 2.5 L<br>↑ 1.9 S
T122	tr4270_c63_g2	12-oxophytodienoate reductase 2 (12-Oxo-PDA-reductase)	Jasmonic acid biosynthesis	↑ 4.0 L
T123	tr4270_c63_g1	12-oxophytodienoate reductase 2 (12-Oxo-PDA-reductase)	Jasmonic acid biosynthesis	↑ 2.8 L<br>↑ 2.5 S
T124	tr4270_c63_g8	12-oxophytodienoate reductase 2 (12-Oxo-PDA-reductase)	Biosíntesis de ácido jasmónico	↑ 2.7 L<br>↑ 3.7 S
T125	tr4270_c63_g13	12-oxophytodienoate reductase 2 (12-Oxo-PDA-reductase)	Jasmonic acid biosynthesis	↑ 2.6 L<br>↑ 4.4 S
T126	tr4270_c63_g10	12-oxophytodienoate reductase 2 (12-Oxo-PDA-reductase)	Jasmonic acid biosynthesis	↑ 8.5 L<br>↑ 6.3 S
T127	tr4062_c0_g2	12-oxophytodienoate reductase 2 (12-Oxo-PDA-reductase)	Jasmonic acid biosynthesis	↑ 2.2 S
T128	tr3678_c490_g1	Type A Regulator Response 3 (ARR3)	Citokinin signalling	↓ -2.6 S
T129	tr3743_c1776_g1	Type A Regulator Response 3 (ARR3)	Citokinin signalling	↓ -1.1 S
T130	tr5015_c629_g1	Type A Regulator Response 3 (ARR3)	Citokinin signalling	↓ -1.9 L
T131	tr7332_c101_g3	Type A Regulator Response 9 (ARR9)	Cytokinin signalling	↓ -1.3 T

T132	tr 3873_c119_g2	KNOTTED1-like homeobox gene 3 (KNAT3)	Regulador transcripcional familia Homeobox	↓ -0.9 S
T133	tr4751_c25_g1	KNOTTED1-like homeobox gene 7 (KNAT7)	Transcriptional regulator. Homeobox family	↓ -1.3 S
T134	tr3574_c196_g1	isopentenyltransferase 5 (IPT5)	Cytokinin biosynthesis	↓ -3.6 L<br>↓ -2.0 S
T135	tr7325_c211_g1	isopentenyltransferase 5 (IPT5)	Cytokinin biosynthesis	↓ -1.5 L
T136	tr5133_c4_g1	UDP-glucosyl transferase 76C1	Cytokinin conjugation	↑ 0.7 S<br>↑ 0.8 S
T137	tr979_c108_g1	UDP-glucosyl transferase 76C2	Cytokinin conjugation	↑ 1.8 S
T138	tr4966_c42_g1	UDP-Glycosyltransferase superfamily protein	Cytokinin conjugation	↑ 1.5 S
T139	tr3691_c13_g1	UDP-glucosyl transferase 85A3	Cytokinin conjugation	↑ 0.8 S
T140	tr2527_c912_g1	UDP-Glycosyltransferase superfamily protein	Cytokinin conjugation	↑ 1.3 S
T141	tr979_c108_g8	UDP-glucosyl transferase 76E1	Cytokinin conjugation	↑ 1.4 L<br>↑ 1.4 S
T142	tr2527_c914_g1	Cytokinin oxidase/dehydrogenase 1 (CKX1)	Cytokinin catabolism	↑ 2.4 S
T143	tr4029_c449_g1	Cytokinin oxidase/dehydrogenase 4 (CKX4)	Cytokinin catabolism	↑ 5.0 S<br>↑ 5.4 S
T144	tr4738_c764_g1	ent-kaurenoic acid hydroxylase (KAO2)	Gibberellin biosynthesis	↓-2.9 S
T145	tr3921_c82_g9	2-oxoglutarate (2OG) and Fe (II)-dependent oxygenase superfamily protein	Gibberellin biosynthesis	↓-5.6 S
T146	tr4278_c39_g28	2-oxoglutarate (2OG) and Fe (II)-dependent oxygenase superfamily protein	Gibberellin biosynthesis	↓-3.0 S
T147	tr937_c208_g2	2-oxoglutarate (2OG) and Fe (II)-dependent oxygenase superfamily protein	Gibberellin biosynthesis	↓-2.7 L
T148	tr937_c235_g1	GA3 oxidase	Gibberellin biosynthesis	↓-2.3 L
T149	tr5854_c399_g1	GA3 oxidase	Gibberellin biosynthesis	↓-1.3 L
T150	tr7292_c143_g1	GA20 oxidase	Gibberellin biosynthesis	↓-1.6 L
T151	tr4738_c658_g1	GA20 oxidase	Gibberellin biosynthesis	↓-2.2 S
T152	tr5846_c486_g1	GA2 oxidase	Gibberellin catabolism	↑ 0.8 L<br>↑ 3.4 S
T153	tr5831_c10_g1	GAs-regulated GASA/GAST/Snakin family protein (GAST1-homolog1)	Gibberellin-regulated family protein	↑ 6.9 S
T154	tr5884_c71_g5	GAs-regulated GASA/GAST/Snakin family protein (GAST1-homolog3)	Gibberellin-regulated family protein	↓-1.4 L
T155	tr3743_c2135_g1	GAs-regulated GASA/GAST/Snakin family protein (GAST1-homolog4)	Gibberellin-regulated family protein	↓-2.2 L<br>↑ 2.7 S
T156	tr7318_c93_g1	GAs-regulated GASA/GAST/Snakin family protein (GAST1-homolog4)	Gibberellin-regulated family protein	↓-1.8 L<br>↑ 5.9 S
T157	tr5846_c761_g1	GAs-regulated GASA/GAST/Snakin family protein (GAST1-homolog5)	Gibberellin-regulated family protein	↑ 1.2 S
T158	tr7316_c167_g1	HVA22 homologue D	Dehydration response	↑ 2.5 L
T159	tr3743_c1615_g1	HVA22 homologue C	Dehydration response	↑ 1.3 L
T160	tr3996_c70_g1	Alcohol deshidrogenasa (ABA2)	ABA biosynthesis	↓-3.0 L<br>↓ 1.6 S
T161	tr4991_c235_g2	UDP-glucosyl transferase 71B5 (UGT71B5)	ABA conjugation	↑ 2.0 L
T162	tr4991_c629_g1	UDP-glucosyl transferase 71B6 (UGT71B6)	ABA conjugation	↑ 1.3 L
T163	tr4991_c638_g1	UDP-glucosyl transferase 71B6 (UGT71B6)	ABA conjugation	↑ 2.8 L<br>↑ 1.7 S
T164	tr4991_c638_g2	UDP-glucosyl transferase 71B6 (UGT71B6)	ABA conjugation	↑ 2.7 L<br>↑ 2.0 S

T165	tr4991_c577_g2	UDP-glucosyl transferase 71B6 (UGT71B6)	ABA conjugation	↑ 1.1 L
T166	tr4991_c526_g1	UDP-glucosyl transferase 71B6 (UGT71B6)	ABA conjugation	↑ 2.2 L
T167	tr3964_c199_g1	AURORA1 (AUR1)	Microtubule-associated protein	↑ 1.9 L
T168	tr5020_c553_g1	MAP65-3	Microtubule-associated protein	↑ 1.6 L

References:

L, leaves; **S**, stem bases (rooting zone)

Table S3. Primers designed for qPCR validation of RNA-seq data.

Gene name	Primers	Sequences (5' → 3')	Amplicon size (pb)
Endoglucanase 1 (Endo1)	*Endo1_F*	GGGGACAACCCAGCCAAGAC	122
	Endo1_R	TTGCACGAGATGCGGCTAGG	
Citokinin dehydrogenase (CitokDes3)	*CitokDes3_F*	CCGTCAGAGAAAAGCCCGGT	106
	CitokDes3_R	CCCAAACCGCAGCAACGAAC	
Ethylene-responsive transcription tactor (ERF003)	*ERF003_F*	TCAAAGAGCGGGTGGGTGGTCA	102
	ERF003_R	TGCTGAACACTGTTGTCTCCTTGGCCT	
Auxin response factors (ARFs) (ARF6)	*ARF6_F*	TGTCGCTGGTTTTCCTCCGGCA	139
	ARF6_R	AGGGAGCCTCACGGTCACCACT	
Gretchen hagen 3 (GH3-1)	*GH3_F*	GTTCCGTTTTGTGTCGCTCT	160
	GH3_R	ACGATCCTGACCGAAATCAC	
1-aminocyclopropane-1-carboxylate oxidase (ACO)	*ACO_F*	AACTCCTCAAGGACGGCAAA	125
	ACO_R	AATAACCCGGTGCTCCACAC	
Fructose-bisphosphate aldolase (FBA)	*FBA_F*	CCTTCTCATTCGGGCGGGCG	128
	FBA_R	GCCCAGAGTGGCCTCGGAGT	
Cellulose Synthase A Catalytic 8 (CSACat8)	*CSACat8_F*	CCATCTCTTTGCCGTCTTCC	136
	CSACat8_R	TATCAGCAGCGTCGTCCACT	
Isoprene Synthase, Chloroplastic-like (ISC-like)	*ISC-like_F*	AGACGCTGTTCGCAGATGGACAT	100
	ISC-like_R	AGCCAGTTCGTTCACGCAGTTGT	

Table S4. Primers designed for the quantification of age-related gene expression.

Gene name	Primers	Sequences (5' → 3')	Amplicon size (pb)
Gretchen hagen 3 (GH3-1)	*GH3_F*	GTTCCGTTTTGTGTCGCTCT	160
	GH3_R	ACGATCCTGACCGAAATCAC	
Auxin response factor (ARF6)	*ARF6_F*	TGTCGCTGGTTTTCCTCCGGCA	139
	ARF6_R	AGGGAGCCTCACGGTCACCACT	
Arabidopsis response regulator 1 (ARR1)	*ARR1_F*	CCCTCCTGTGATTGGGCTGC	107
	ARR1_R	ATCCTGTTGCCGTGCTCCAC	
Transport Inhibitor Response 1 (TIR1)	*TIR1_F*	GCATGTAAGCTGCTTGGTCA	153
	TIR1_R	AAAATCAGGCATGTCGAACC	
Tryptophan aminotransferase in Arabidopsis 1 (TAA1)	*TAA1_F*	GGGAACAGGTGACAAACTCAA	157
	TAA1_R	GGCTTACATGGACCTGAAGA	
Scarecrow-Like 1 (SCL1)	*SCL1_F*	AGGCGCTCTTCCATTATTCG	130
	SCL1_R	CCTTCGCAGGCAATTACGTT	
Aintegumenta-Like 1 (AIL1)	AIL1_*F*	AGGCACTGGCGTTGGTGGTG	142
	AIL1_R	AGATGGCCGGGTCCCCCAAG	
